# The utility of DNA barcodes to confirm the identification of palm collections in botanical gardens

**DOI:** 10.1371/journal.pone.0235569

**Published:** 2020-07-31

**Authors:** Duc-Thanh Le, Yu-Qu Zhang, Yong Xu, Li-Xiu Guo, Zhi-Ping Ruan, Kevin S. Burgess, Xue-Jun Ge

**Affiliations:** 1 Key Laboratory of Plant Resources Conservation and Sustainable Utilization, South China Botanical Garden, Chinese Academy of Sciences, Guangzhou, China; 2 College of Life Sciences, University of Chinese Academy of Sciences, Beijing, China; 3 Xiamen Botanical Garden, Xiamen, China; 4 Department of Biology, Columbus State University, University System of Georgia, Columbus, Georgia, United States of America; National Cheng Kung University, TAIWAN

## Abstract

The palm family (Arecaceae) is of high ecological and economic value, yet identification in the family remains a challenge for both taxonomists and horticulturalists. The family consists of approximately 2600 species across 181 genera and DNA barcoding may be a useful tool for species identification within the group. However, there have been few systematic evaluations of DNA barcodes for the palm family. In the present study, five DNA barcodes (*rbcL*, *matK*, *trnH*-*psbA*, ITS, ITS2) were evaluated for species identification ability across 669 samples representing 314 species and 100 genera in the Arecaceae, employing four analytical methods. The ITS gene region was found to not be a suitable barcode for the palm family, due in part, to low recovery rates and paralogous gene copies. Among the four analyses used, species resolution for ITS2 was much higher than that achieved with the plastid barcodes alone (*rbcL*, *matK*, *trnH*-*psbA*), and the barcode combination ITS2 + *matK* + *rbcL* gave the highest resolution among all single barcodes and their combinations, followed by ITS2 + *matK*. Among 669 palm samples analyzed, 110 samples (16.3%) were found to be misidentified. The 2992 DNA barcode sequences generated in this study greatly enriches the existing identification toolbox available to plant taxonomists that are interested in researching genetic relationships among palm taxa as well as for horticulturalists that need to confirm palm collections for botanical garden curation and horticultural applications. Our results indicate that the use of the ITS2 DNA barcode gene region provides a useful and cost-effective tool to confirm the identity of taxa in the Palm family.

## Introduction

Botanical gardens typically hold a wide diversity of well-documented living plant collections for the purpose of scientific research, conservation, display and education. Globally, botanical gardens conserve at least 41% of known threatened plant species in their living collections and seed banks [1]. Although most botanical gardens are curated by taxonomic experts, which often specialize in specific groups of plant taxa, there is often a considerable percentage of botanical garden collections that are often misidentified or not resolved to the species, or even, genus level. Many plants grown in botanical gardens have been obtained as seed that are either misidentified during collection from wild or cultivated sources, or have had their identity lost or reassigned during the cultivation process within the garden. To add to the problem, which may be especially poignant for small botanical gardens that are understaffed or display focused, herbarium vouchers and taxonomic experts are often lacking.

A group of taxa for which species identification at botanical gardens may be particularly problematic are the Palms (Arecaceae). The Arecaceae is composed of 181 genera (approximately 2600 species) that are concentrated primarily in moist equatorial, tropical and subtropical regions [2, 3]. The economic and horticultural importance of palms ranks them third among the most important plant families for human use, following grasses and legumes [4]; the fruits are the primary food source for many indigenous peoples as well as numerous vertebrates. In addition, almost all palm species are commonly used as ornamentals and many are economically important species: coconut (*Cocos nucifera* L.), the African oil palm (*Elaeis guinensis* Jacq.), date palm (*Phoenix dactylifera* L.) and the Saw Palmetto (*Serenoa repens* Small), to name a few. Despite the importance of palms to the economy and many ecosystems, accurate morphological identification of palm species, especially at the seedling stage, remains a challenge for taxonomists and gardeners at botanical gardens.

The height of some species, and their large leaves and/or thorny characteristics, make species classification and identification based on herbarium specimens difficult for palms. The specimens are usually only part of the entire plant, being selected from leaves and inflorescences, if available. In addition, floral morphology can change dramatically among different developmental stages and identification at the seedling stage is difficult due to the similarity of morphological characters. Due to the lack of taxonomic expertise on this family and the large number of known palm species, failure to identify species, or misidentification, is not uncommon in botanical garden collections.

DNA barcoding may be a particularly valuable tool for confirming the identification of palm species, especially for specimens at immature stages of development, where diagnostic floral characteristics are rarely present in many botanical garden collections. Despite the species richness and economic or cultural importance of the palm family, there have only been a handful of studies that have utilized DNA barcoding to resolve species relationships in the group [5–7], although rates of species discrimination based on DNA barcoding varies among studies and genera. For example, among 40 out of the 48 species of the southeast Asian tribe Caryoteae, two DNA barcodes (*rbcL* and *matK*) revealed relatively low species discrimination rates, and ITS2 was chosen over *trnH-psbA* as a supplemental region to these two ‘core’ markers [5]. In contrast, in a study on 15 Chinese *Calamus* species [6], *trnH*-*psbA* was recommended as an appropriate single DNA barcode, and ITS was eliminated from consideration due to low sequence recovery rates, and the presence of paralogous sequences. Previous molecular phylogenetic studies based on or including plastid data (including *matK*, *rbcL*, *rps16* and *trnL*-*trnF*) have also demonstrated low sequence variation within the palm family (e.g., [8–11]). Given the discrepancy among previous studies, a comprehensive DNA barcode study of the palms from across an extensive sampling range is important if the approach is to be applied for the confirmation of palm taxa identifications at botanical gardens.

To determine the utility of DNA barcodes to confirm the genetic identity of palms in botanical garden collections, we sampled more than 300 palm species cultivated at three botanical gardens in China. To address this goal, we 1) evaluate taxon resolution for individual barcodes and well as in combination and 2) determine rates of identification failures found in existing botanical collections.

## Materials and methods

### Ethics statement

The South China Botanical Garden, the Xiamen Botanical Garden and the Xishuangbanna Tropical Botanical Garden granted permission for palm samples collection.

### Taxon sampling

Voucher specimens and DNA samples were collected from three botanical gardens in China, viz. the South China Botanical Garden (SCBG) at Guangzhou, the Xiamen Botanical Garden (XMBG) at Xiamen and the Xishuangbanna Tropical Botanical Garden (XTBG) at Jinghong, Yunnan. These gardens harbor the most prominent collections of palms in China. Young leaves were stored in silica gel for DNA analysis. A total of 669 samples from 314 species across 100 genera were collected (S1 Table). All voucher specimens were deposited in the Herbarium of the South China Botanical Garden, Chinese Academy of Sciences (IBSC).

The classification system followed Baker & Dransfield [3] and http://powo.science.kew.org/. Plant identifications were compared with online libraries of images of living plants, monographs such as Genera Palmarum [2], and an encyclopedia of cultivated palms [12].

### DNA extraction, amplification and sequencing

Total DNA was extracted from dried leaf tissue using a CTAB method [13]. The amplification of *rbcL*, *matK*, *trnH-psbA*, ITS and ITS2 was carried out with universal primer sets ([14–19], Table 1). We amplified DNA in a 25 μL reaction mixture following Zhang et al. [20] using *rTaq* DNA polymerase. For those samples that failed to amplify on a first pass, LA or Primer Star DNA polymerase (Takara Biotechnology Co. Ltd.) or 2*T5 Super PCR Mix (Beijing TsingKe Biotech Co., Ltd.) was used as an alternative to *rTaq* DNA polymerase. Samples showing a clear single band were sent to Shanghai Majorbio Bio-Pharm Technology Co., Ltd., Shanghai, China for bi-directional sequencing. All sequences were uploaded to the GenBank (GenBank accession numbers are given in S1 Table).

**Table 1:** Primers used for amplification and sequencing of five single markers.

Region	Primer names	Primer sequences (5′-3′)	References
***rbcL***	*rbcLa* F	ATGTCACCACAAACAGAGACTAAAGC	Kress *et al*. [14]
	*rbcLa* R	GTAAAATCAAGTCCACCRCG	
***matK***	KIM-3F	CGTACAGTACTTTTGTGTTTACGAG	Kim, unpublished
	KIM-1R	ACCCAGTCCATCTGGAAATCTTGGTTC	
***trnH-psbA***	*psbA3*	GTTATGCATGAACGTAATGCTC	Sang *et al*. [15]
	*trnH05*	CGCGCATGGTGGATTCACAATCC	Tate & Simpson [16]
**ITS**	ITS-leu1	GTCCACTGAACCTTATCATTTAG	Urbatsh *et al*. [17]
	ITS4	TCCTCCGCTTATTGATATGC	White *et al*. [18]
**ITS2**	ITS2 S2F	ATGCGATACTTGGTGTGAAT	Chen *et al*. [19]
	ITS2 S3R	GACGCTTCTCCAGACTACAAT	

### Data analysis

Raw sequences were assembled and edited using Geneious v.10.2.3 [21]. Edited sequences were then aligned using the default option implemented in MAFFT [22] as a plugin in Geneious [21]. Inversions in *trnH-psbA* were edited manually following Jeanson et al. [5]. We evaluated sixteen DNA barcodes, which included five single loci and eleven combinations using the following methods. Firstly a genetic distance-based method was used and based on two analyses: (a) The values of intra- and inter-specific divergence were calculated using the Kimura 2-parameter (K2P) distances in MEGA 7.0.26 [23]. To detect barcode gaps, we used both histogram and scatter plot approaches. Histograms were generated from the distribution of divergence at intervals of 0.005 distance units, based on the “pairwise summary” function in the program TaxonDNA [24]. Scatter plots were compiled using R version 3.2.5 [25], with each dot representing a species; the values of intra-specific and inter-specific distances for each species were calculated with the “extreme pairwise” function in the program TaxonDNA [24]. We then searched for the minimum inter-specific distance and maximum intra-specific distance for each species using a custom R script [25]; and (b) Unrooted Neighbor-Joining (NJ) trees were constructed in MEGA 7.0.26 [23], with pairwise deletion based on the *P*-distance model [26]. The calculation of node support was based on 1000 bootstrap replicates. A species was considered to have been successfully identified only when all conspecific individuals formed a single clade with a bootstrap value ≥50% [27]. Secondly, we used a tree-based method, where Maximum likelihood (ML) trees based on the GTR + GAMMA substitution model and 1000 bootstrap replicates were reconstructed using RAxML-HPC2 v8.2.12 [28] in the CIPRES Science Gateway [29]. If conspecific (congeneric) sequences formed a monophyletic clade with bootstrap support of 50% or greater [30], we considered that species (genus) to be correctly identified. Finally, a similarity-based method based on the “Best match” (BM) and “Best close match” (BCM) functions in the program TaxonDNA [24] was used to calculate percentage identification success [24].

### Species confirmation

During voucher collection at each garden, all samples were photographed and the species identification label was noted, and subsequently verified using traditional taxonomical methods and comparisons to the online image library of living plants and monographs. These "traditional" palm identifications were then compared to identifications based on DNA barcodes. Because the barcode combination (ITS2 + *matK + rbcL*) achieved the highest rate of species resolution in NJ-tree analysis, the NJ-tree of this combination was used for species confirmation. The barcode sequences were a composite of barcodes from the barcode library established in this study and those downloaded from GenBank. For those samples with different a species' name, yet clustering within a clade having a bootstrap value higher than or equal to 50%, specimens were rechecked in order to verify whether they were misidentified and subsequently changed to the correct name.

A total of 2098 *rbcL* sequences, 1504 *matK* sequences, 783 ITS/ITS2 sequences, and 723 *trnH-psbA* sequences were downloaded from GenBank and extracted from the complete chloroplast genome available on July 10th, 2019. The downloaded barcode sequences from GenBank were filtered. We then removed sequences shorter than 300 bp in length (for *rbcL*, *matK*, and ITS) or shorter than 200 bp in length (for ITS2 and *trnH-psbA*), of poor quality, or with the species name within the genus unspecified. Synonyms and incorrect names were corrected according to the website http://powo.science.kew.org/, with the names of palm genera following Baker and Dransfield [3]. After filtering, there were 1563 *rbcL* sequences from 427 species (176 genera), 1197 *matK* sequences from 571 species (170 genera), 293 ITS2 sequences from 147 species (42 genera), 432 ITS sequences from 174 species (44 genera), and 718 *trnH-psbA* sequences of 162 species (45 genera). Due to the highly variable sequence length and alignment difficulty for *trnH-psbA*, we did not use *trnH-psbA* sequences for species confirmation. In addition, the paralogous copies that we found in many of the ITS sequences rendered this gene region unsuitable for palm species identification. In total, our final database contained 2232 *rbcL* sequences from 562 species (177 genera), 1865 *matK* sequences from 671 species (173 genera), and 919 ITS2 sequences from 385 species (108 genera).

## Results

### Barcode recovery

A total of 2,992 new barcode sequences (669, 668, 660, 626 and 369, for *rbcL*, *matK*, *trnH*-*psbA*, ITS2, and ITS, respectively) were obtained from 669 samples representing 314 species and 100 genera in the Arecaceae. All sequences were submitted to the NCBI database (S1 Table). The ITS gene region had the lowest percentage sequencing success (55.2%), whereas the other four barcodes showed relatively high success rates, which ranged from 100% (*rbcL*) to 93.6% (ITS2). For *rbcL*, *matK*, *trnH-psbA* and ITS2, a database containing a subset of 617 sequences per barcode was used for further investigation. In this database, there were 431 sequences from 151 species with more than one individual per species. The ITS gene region was analyzed separately because the number of sequences available for this barcode was much lower than that for the other four barcodes.

Aligned barcode lengths varied from 538 bp (*rbcL*) to 1735 bp (*trnH-psbA*) (Table 2). ITS and ITS2 had the highest percentages of variable sites (79.0% and 71.4%, respectively) and parsimonious-informative characters (68.1% and 65.1%, respectively), while *rbcL*, *matK* and their combination had the lowest (variable sites: 12.5%, 28.0%, and 22.0%, respectively; parsimonious-informative sites: 11.2%, 22.8%, and 18.3%, respectively) (Table 2). Due to a high level of sequence length variation (ranging from 353 bp to 1061 bp), *trnH-psbA* could not be aligned; this intergenic spacer is more variable than *rbcL* and *matK* (variable sites: 31.6%, 12.5%, and 28.0% respectively; parsimonious-informative sites: 26.4%, 11.2%, and 22.8% respectively). The mean pairwise inter-specific distance was lowest for *rbcL* (0.0080) and highest for ITS (0.3070). ITS exhibited the highest mean intra- and inter-specific distances (0.0364, 0.3070), followed by ITS2 (0.0013, 0.1532) and *trnH-psbA* (0.0011, 0.0306), while *matK* (0.0002, 0.0190) and *rbcL* (0.0001, 0.0080) had the lowest (Table 2).

**Table 2:** Characteristics of the five single markers and eleven combinations evaluated in this study.

Barcode	Aligned length (bp)	No. of variable sites (%)	No. of parsimony-informative sites (%)	Mean intraspecific distance	Minimum intraspecific distance	Maximum intraspecific distance	Mean interspecific distance	Minimum interspecific distance	Maximum interspecific distance
R	538	67 (12.5)	60 (11.2)	0.0001	0	0.0037	0.0080	0	0.0265
M	846	237 (28.0)	193 (22.8)	0.0002	0	0.0062	0.0190	0	0.0671
I	1254	990 (79.0)	854 (68.1)	0.0364	0	0.2382	0.3070	0	0.6057
I2	657	469 (71.4)	428 (65.1)	0.0013	0	0.0208	0.1532	0	0.3937
T	1735	549 (31.6)	458 (26.4)	0.0011	0	0.0284	0.0306	0	0.1765
MR	1384	304 (22.0)	253 (18.3)	0.0001	0	0.0037	0.0145	0	0.0483
I2R	1195	536 (44.9)	488 (40.8)	0.0007	0	0.0096	0.0714	0	0.1609
RT	2273	616 (27.1)	518 (22.8)	0.0006	0	0.0156	0.0197	0	0.0968
I2M	1503	706 (47.0)	621 (41.3)	0.0006	0	0.0097	0.0643	0	0.1381
MT	2581	786 (30.5)	651 (25.2)	0.0006	0	0.0127	0.0239	0	0.0944
I2T	2392	1018 (42.6)	886 (37.0)	0.0012	0	0.0171	0.0822	0	0.2612
I2MR	2041	773 (37.9)	681 (33.4)	0.0004	0	0.0068	0.0475	0	0.0992
I2MT	3238	1255 (38.8)	1079 (33.3)	0.0008	0	0.0099	0.0538	0	0.1396
MRT	3119	853 (27.3)	711 (22.8)	0.0004	0	0.0093	0.0194	0	0.0708
I2RT	2930	1085 (37.0)	946 (32.3)	0.0008	0	0.0116	0.0561	0	0.1496
I2MRT	3776	1322 (35.0)	1139 (30.2)	0.0006	0	0.0078	0.0433	0	0.1063

*rbcL (R); *matK* (M); ITS (I); ITS2 (I2); *trnH-psbA* (T).

### Taxon resolution

For the genetic distance method (based on histograms), no distinctive barcode gaps were detected for any of the markers, whereas barcoding gaps were revealed using the scatter plot analysis (S1 Fig). Among single barcodes, ITS2 (75.8%) showed the highest species resolution, followed by *trnH-psbA* (53.9%), with *matK* and *rbcL* showing lower rates of species resolution (35.2% and 14.8%, respectively). Of the eleven combinations, ITS2 + *matK* + *rbcL* exhibited the highest species resolution (83.6%), followed by ITS2 + *matK* (81.3%), ITS2 + *rbcL* (80.5%) and ITS2 + *rbcL* + *trnH*-*psbA* (77.3%) (Table 3). For the genetic distance method based on the NJ-tree analysis the same patterns were found. ITS2 + *matK* + *rbcL* had the highest species resolution (89.4%, Table 3, S2 Fig) among all single and combined barcodes, followed by ITS2 + *matK* (86.8%) and ITS2 + *rbcL* (84.1%) (Table 3). For individual barcodes, ITS2 had the highest percentage resolution (species: 82.8%, genus: 90.5%) (Table 3, S3 Fig). The plastid barcodes (*rbcL*, *matK* and *trnH-psbA*) demonstrated relatively low resolution; (species: 13.2%, 35.1%, 42.4%; genus: 21.6%, 64.9%, 50.0%) (Table 3).

**Table 3:** Identification success rates obtained using distance and tree methods for five single markers and eleven combinations.

DNA region	Distance method—Species level (%)	NJ tree method–Species level (%)	NJ tree method—Genus level (%)	ML tree method–Species level (%)	ML tree method–Genus level (%)
*rbcL*	14.8	13.2	21.6	14.6	21.6
*matK*	35.2	35.1	64.9	38.4	62.2
ITS	50.0	64.9	84.0	62.8	71.7
ITS2	75.8	82.8	90.5	82.8	91.9
*trnH-psbA*	53.9	42.4	50.0	45	48.6
*matK + rbcL*	42.2	44.4	77.0	45	73
ITS2 + *rbcL*	80.5	84.1	90.5	84.1	91.9
*rbcL* + *trnH-psbA*	40.6	47.7	59.5	48.3	58.1
ITS2 + *matK*	81.3	86.8	93.2	86.1	93.2
*matK* + *trnH-psbA*	46.9	51.7	71.6	53.6	70.3
ITS2 + *trnH-psbA*	74.2	78.1	85.1	80.1	89.2
ITS2 + *matK* + *rbcL*	83.6	89.4	93.2	88.1	94.6
ITS2 *+ matK* + *trnH-psbA*	75.8	82.1	86.5	80.1	90.5
*matK* + *rbcL* + *trnH-psbA*	50.8	54.3	74.3	55	74.3
ITS2 + *rbcL* + *trnH-psbA*	77.3	79.5	87.8	78.1	90.5
ITS2 + *matK* + *rbcL* + *trnH-psbA*	77.3	82.8	93.2	80.1	90.5

For the ML tree-based method, ITS2 (82.8%) and the combination ITS2 + *matK* + *rbcL* (88.1%) revealed the highest species resolution among single barcodes and their combinations, respectively (Table 3). The "core" barcode *matK* + *rbcL* recommended by CBOL had relatively low species resolution (45%). At the genus level, ITS2 *+ matK + rbcL* had the highest resolution (94.6%) among all the barcodes (and their combinations), and was higher than that of the NJ tree analysis (93.2%) (Table 3). Five of the 74 genera with more than one sample were not found to be monophyletic (*Astrocaryum*; *Brahea*; *Kentiopsis*, *Syagrus*) (S2 and S4 Figs) based on the three barcode combination ITS2 *+ matK + rbcL*.

For the similarity-based method, similar results were obtained for the BM model and the BCM model (Table 4). Among the eleven barcode combinations, ITS2 + *matK* + *rbcL* had the highest percentage species resolution for each respective model (88.7%, 88.7%), followed by ITS2 + *matK* (86.8%, 86.8%), ITS2 + *rbcL* (85.4%, 85.4%), ITS2 + *matK* + *rbcL* + *trnH*-*psbA* (83.4%, 82.8%) and ITS2 + *matK* + *trnH*-*psbA* (82.1%, 81.5%) (Table 4). Among the five single barcodes, the ITS2 had the highest rate (80.8%, 80.8%), followed by *trnH-psbA* (60.3%, 59.6%), *matK* (38.4%, 38.4%) and *rbcL* (14.6%, 14.6%).

**Table 4:** Identification success rate based on the similarity method using ‘best match’ and ‘best close match’ models in the TaxonDNA program.

DNA region	Best match (%)			Best close match (%)			
	Correct	Ambiguous	Incorrect	Correct	Ambiguous	Incorrect	Outside
*rbcL*	14.6	82.8	2.6	14.6	82.8	2.6	0
*matK*	38.4	54.3	7.3	38.4	54.3	6.6	0.7
ITS	57.7	2.6	39.7	57.7	2.6	35.9	3.8
ITS2	80.8	15.2	4	80.8	15.2	4	0
*trnH-psbA*	60.3	21.9	17.9	59.6	21.9	15.2	3.3
*matK + rbcL*	47.0	46.4	6.6	47	46.4	6	0.7
ITS2 + *rbcL*	85.4	11.3	3.3	85.4	11.3	3.3	0
*rbcL* + *trnH-psbA*	47.0	38.4	14.6	47	38.4	14.6	0
ITS2 + *matK*	86.8	11.2	2	86.8	11.2	2	0
*matK* + *trnH-psbA*	54.3	29.1	16.6	54.3	29.1	15.9	0.7
ITS2 + *trnH-psbA*	80.8	10.6	8.6	80.1	10.6	8.6	0.7
ITS2 + *matK* + *rbcL*	88.7	9.3	2	88.7	9.3	1.3	0.7
ITS2 *+ matK* + *trnH-psbA*	82.1	9.9	7.9	81.5	9.9	7.9	0.7
*matK* + *rbcL* + *trnH-psbA*	58.3	26.5	15.2	58.3	26.5	14.6	0.7
ITS2 + *rbcL* + *trnH-psbA*	82.8	10.6	6.6	82.1	10.6	6.6	0.7
ITS2 + *matK* + *rbcL* + *trnH-psbA*	83.4	9.9	6.6	82.8	9.9	6.6	0.7

### Species confirmation

Because the highest level of species resolution was found for ITS2 + *matK* + *rbcL* (NJ-tree analysis; 89.4%, Table 3), we used this combination to screen the identification of the samples collected from the three Chinese botanical gardens. Among the 669 palm samples used for this analysis, 110 samples (16.4%) were found as misidentified. Among these, 90 samples were misidentified at the species level, and 20 samples were misidentified at the genus level (S3 Table).

## Discussion

Construction of DNA barcode reference databases for tropical plants is still a challenge for the plant DNA barcoding community. Despite the high economic importance of palms, there are relatively few DNA barcodes available in the NCBI GenBank database and there are few studies on barcoding in palms. As of July 10th, 2019, GenBank database contained 5108 Arecaceae sequences for the five DNA barcode regions analyzed in this study, 70.5% (3602 sequences) are for *rbcL* and *matK* and after filtering, only 2760 were found to be of high quality. Among the remainder, 725 sequences are ITS and ITS2. Our study contributes nearly 3,000 sequences across 100 palm genera (S1 Table), and significantly enriches this database with ITS2 sequences (626 sequences). The development of this now more comprehensive barcode library will be a valuable resource for a wide range of future applications, including species identification and confirmation, systematic and phylogenetic studies, conservation programs, ecological research, and the confirmation of species for the palm industry.

### Evaluation of DNA barcodes for the palm family

The "core" plant DNA barcodes, *rbcL* and *matK*, suggested by the CBOL Plant Working Group [31] exhibited relatively low rates of species discrimination for the Arecaceae, both individually and in combination across all four of the different analytical methods used in the present study (13.2%-47.0%) (Tables 3, 4). This result is consistent with those for the Caryoteae [5] and *Calamus* [6] and previous molecular phylogenetic studies have also revealed that *rbcL* and *matK* are unusually, highly conserved in palms compared to other monocots (e.g., [4, 8, 10, 32]). These relatively low species discrimination rates may be partly attributed to the long generation time of the Arecaceae [33–35]. In addition, the efficacy of DNA barcoding to identify species is dependent on species that are monophyletic [36], yet in many cases non-monophyletic species have been reported for the palms. For example, the three widely distributed Neotropical palm species *Euterpe precatoria*, *Hyospathe elegans*, and *Prestoea acuminata* are non-monophyletic [37] and in our study, non-monophyletic species were found in several genera (i.e., *Arenga*, *Butia*, *Coccothrinax*, *Phoenix*, *Ptychosperma*, *Livistona*, *Sabal*, *Thrinax*) (S2 and S3 Figs) from all the barcodes and analysis methods. Although paraphyletic or polyphyletic species may be one reason for low discrimination rates found in our study, low rates even at the genus level (21.6%-77.0%; Table 3), certainly indicates the core barcodes, *rbcL* and *matK* are not suitable for the confirmation of palm species at botanical gardens.

The chloroplast gene region *trnH-psbA* has been proposed as supplementary barcodes for many plant taxa [19, 38, 39]. However, we found many intra- and inter-specific micro-inversions and indels in several of the palm species that we studied, a finding that is in-line with previous studies that have also demonstrated that *trnH-psbA* has considerable interspecific variation, and even intraspecific variation, including the presence of inversions and insertion-deletion polymorphisms (indels) [40, 41]. The original length of *trnH*-*psbA* in our study varied from 353 bp to 1061 bp, however, the high occurrence of indels caused the aligned length to be 1735 bp. Manually correcting these inversions, insertions and/or deletions and then attempting to align the *trnH-psbA* spacer region is a widely-observed, labor-intensive protocol that requires careful visual inspection during the alignment process. Although *trnH-psbA* demonstrated higher discriminatory performance than *matK* and *rbcL* in our study, its resolution was also found to be much lower than ITS2 for many of our palm taxa (Tables 3 and 4). Considering the limited number of high-quality sequences for palm species on GenBank, the limited discrimination power associated with this gene region, and the issues associated with aligning this region among disparate species, we support the suggestion of Jeanson et al. [5] that *trnH-psbA* should not be used for the confirmation of palm identifications at botanical gardens.

The ITS gene region has yielded relatively high levels of species resolution in many DNA barcode evaluation studies [19, 39]. However, in our study, sequence recovery was low (55.2%), even when using different *Taq* DNA polymerases and additional primer sets; this problem has also been shown for *Calamus* ([6], 25% PCR success rates). In contrast to the lack of sequence recovery for ITS, ITS2 (93.6%) was much easier to amplify and sequence than the entire region, a result also found in numerous studies across a broad range of taxa [42]. For the palm taxa analyzed in our study, ITS2 provided higher taxa resolution than plastid barcodes, which also increased when ITS2 was combined with the two DNA barcodes *rbcL* and *matK* (Tables 2–4). In most taxa, species resolution for ITS2 is often higher than that of the plastid regions, especially for closely-related species (e.g. [27, 43]). Due to the high degree of universality of its primers, its short sequence length and high capacity for species resolution, ITS2 has been widely used in plant barcoding [44, 45], especially in metabarcoding in recent years [46]; e.g., for pollen provenance determination [47] and for environmental DNA identification [48]. Taking into account the high rate of species resolution and the cost-effectiveness associated with the relatively high sequence recovery rates, we agree with Jeanson et al. [5] that ITS2 should be to supplement the two ‘core’ markers in palms, which has been shown to have consistent results across a range of families and genera (e.g., [49]). In addition and given that divergent paralogues and multiple PCR bands were observed for the entire ITS gene region in our study and that recovery is certainly an important criterion for the development of a cost-effective DNA barcoding strategy [42], we also agree with Yang et al. [6] that entire ITS gene region is not a suitable barcode for the confirmation of palm collections at Botanical Gardens.

The addition of ITS2 to combinations of plastid markers greatly increased the species resolution rates found in our study (Tables 3 and 4). In particular, the combination of ITS2 + *matK* + *rbcL* demonstrated the highest discriminatory rate among the eleven combinations analyzed (Tables 3 and 4), this combination has also been used successfully as a standard DNA barcode in other floristic studies, e.g., [50]. At genus level, however, five of the 74 genera with more than one sample were not recovered as a monophyletic clade (*Astrocaryum*, *Brahea*; *Dypsis*; *Kentiopsis*, *Syagrus*) based on the NJ tree (S2 Fig) or the ML tree (S4 Fig) of the combination ITS2 + *matK* + *rbcL*. Among these five genera, *Astrocaryum* [51], *Brahea* [52] and *Syagrus* [51] have been shown to be monophyletic in previous studies, while the monophyly of *Dypsis* and *Kentiopsis* was not supported [53–55]. It is possible that erroneous topologies may be obtained when the data are not informative [56] and this may indeed be the case for *Astrocaryum* and *Brahea*, where a lack of monophyly may be due to the low resolution of the markers that we used, where ITS (for *Astrocaryum*) and combinations with *trnH-psbA* (I2T, MT, RT, I2MT, I2RT, MRT, I2MRT for *Brahea*) were found to be monophyletic in our study. In addition, for the genus *Syagrus*, which we found to form a clade with the closely related, monotypic genus *Cocos* [51, 57], previous studies have separated the two genera based on six WRKY gene-family loci [51]. Notably, *Dypsis* and *Kentiopsis* could not be recovered as monophyletic based on all barcodes studied, which is in line with other studies [53–55], although it has been suggested that the large and variable genus *Dypsis* be divided into several smaller genera [54, 55]. Considering the large number of *rbcL* and *matK* sequences currently deposited in NCBI GenBank database, coupled with the overall discrimination ability of their combination with ITS2, we suggest ITS2 +*matK* + *rbcL* may serve as an effective molecular tool for the confirmation of palm identifications at botanical gardens.

### Species resolution in complex genera

Many factors can influence species resolution where, in addition to their evolutionary history, the number of species in a genus can lower discrimination rates in species-rich genera [58]. In the palm family, ca. 70% of the species belong to 42 genera that have more than 10 species each (http://powo.science.kew.org/). As such, these genera pose a significant challenge for DNA barcoding identification of palms in botanical gardens. In the present study, the combination of ITS2 + *matK* + *rbcL* identified between 66.7% -100% of the species among seven of the eight large genera that we studied (S2 Table). This unexpected, high degree of species resolution may be due to the distant evolutionary relationships among the few species that were sampled. For example, the seven *Chamaedorea* species studied (S1 Table) are from four different subgenera [59]. Alternatively, many DNA barcoding or phylogenetic studies have demonstrated that it is difficult to identify species from those genera that have undergone rapid radiation [60–62]. *Sabal* is a genus with 18 species, and due to the low degree of divergence among *Sabal* species, the phylogenetic tree estimated from the plastome sequences shows low species resolution and low support values [63]. This may explain why only two of the six *Sabal* species in the present study could be identified (S2 Table). For these reasons, DNA barcoding of palm taxa, may still possess some significant challenges in complex genera that are species rich, although the barcode library developed in this study will contribute to the identification toolkit available for the future curation of botanical garden palm collections.

### Application of DNA barcodes for species identification

In the present study, 110 samples (16.4%) were found to be misidentified, and most were at the species level (90 out of 110 misidentified samples) (S3 Table), a result that is not negligible given that most botanical gardens serve as an important genetic resource (seeds, propagules, cuttings) for both scientific and horticultural applications. Although botanical gardens usually have taxonomic experts for different taxonomic groups, this result indicates that DNA barcoding could serve as a significant tool for the confirmation of palm identifications for important collections at botanical gardens. Most palm species grown within Chinese botanical gardens are imported from other countries through plant (seed) catalogues or inventory lists. During seed collection, prior to shipment to botanical gardens, it seems reasonable to assume that lack of documentation and unregulated trade practices can result in the mislabeling or misidentification of palm taxa, a process that can certainly result in downstream errors that cumulate and spread over time. Our study indicates that DNA barcoding technology may be a powerful tool for species confirmation, and in turn, the effective curation of botanic garden palm collections.

## Conclusion

Construction of DNA barcode reference databases is still a challenge, especially for plant families from tropical regions. To satisfy the high demand for accurate species identification in the palm family, an increase in the number of DNA barcode **s**equences, in terms of both taxon coverage and the number of ITS2 sequences in particular, is greatly needed. The 2992 DNA barcode sequences generated in this study greatly enriches the existing identification toolbox available to plant taxonomists that are interested in researching genetic relationships among palm taxa as well as for horticulturalists that need to confirm palm collections for botanical garden curation and horticultural applications. To increase the discriminatory power for genera that contain a large number of species, the use of 2^nd^ generation DNA barcodes has been proposed, focusing on either the capture of nuclear gene markers or genome skimming [64]. The availability of full plastid genomes could increase species resolution dramatically [65]. However, it is still an expensive approach for use on a large scale and at institutions that are struggling for funding. Our results indicate that the use of the ITS2 DNA barcode gene region, and where possible its combination with *matK* + *rbcL*, will provide a useful and cost-effective molecular tool to confirm the genetic identity of botanical garden palm collections.

## Supporting information

S1 Table Specimen voucher numbers and GenBank accession numbers of five markers for all samples used in this study. (DOCX)

S2 Table The species resolution of the eight large genera studied (≥ 5 species per genus). (DOCX)

S3 Table Misidentified samples in the botanical garden. (DOCX)

S1 Fig Scatter plots of the maximum intra-specific K2P distance versus minimum inter-specific K2P distance for five single markers and eleven combinations (Note: I, internal transcribed spacer (ITS); I2, ITS2; M, *matK*; R, *rbcL*; T, *trnH-psbA*). (PDF)

S2 Fig Neighbor-Joining (NJ) tree generated using the combined sequences of ITS2 + *matK* + *rbcL*. Bootstrap values are shown above the relevant branches.
(PDF)

S3 Fig Neighbor-Joining (NJ) tree generated using ITS2 sequences. Bootstrap values are shown above the relevant branches.
(PDF)

S4 Fig A Maximum likelihood (ML) tree generated using the combined sequences of ITS2 + *matK* + *rbcL*. Bootstrap values are shown below the relevant branches.
(PDF)
