# Supplementary material for: The utility of DNA barcodes to confirm the identification of palm collections in botanical gardens
Source: PLoS One. 2020 Jul 31;15(7):e0235569. doi: 10.1371/journal.pone.0235569 (PMC7394517; doi:10.1371/journal.pone.0235569)
Supplement: S1 Table — (DOCX) [file pone.0235569.s001.docx]

| **Table S1.** Specimen voucher numbers and GenBank accession numbers of five markers for all samples used in this study | | | | | | | |
| --- | --- | --- | --- | --- | --- | --- | --- |
| **No.** | **Species** | **Specimen voucher** | ***rbcL*** | ***matK*** | **nrITS2** | ***psbA–trnH*** | **nrITS** |
| 1 | *Acoelorrhaphe wrightii* | SCBG014 | MK753389 | MK704640 | MK691917 | MK756349 | \\\ |
| 2 | *Acrocomia crispa* | XTBG215 | MK753771 | MK704748 | MK691802 | MK756557 | MK683211 |
| 3 | *Adonidia merrillii* | XTBG045 | MK753635 | MK704941 | MK692217 | MK756958 | MK683274 |
| 4 | *Aiphanes eggersii* | XMBG175 | MK753778 | MK704753 | MK691808 | MK756670 | MK683197 |
| 5 | *Aiphanes eggersii* | XMBG178 | MK753779 | MK704754 | MK691809 | MK756672 | MK683198 |
| 6 | *Aiphanes eggersii* | XTBG209 | MK753780 | MK704755 | MK691810 | MK756671 | MK683199 |
| 7 | *Aiphanes horrida* | XMBG100 | MK753782 | MK704757 | MK691815 | MK756673 | \\\ |
| 8 | *Aiphanes horrida* | XTBG235 | MK753783 | MK704758 | MK691816 | MK756674 | MK683201 |
| 9 | *Aiphanes sp.* | XMBG101 | MK753856 | MK704759 | MK691814 | MK756675 | MK683200 |
| 10 | *Aiphanes sp.* | XTBG198 | MK753781 | MK704756 | MK691811 | MK756676 | MK683202 |
| 11 | *Allagoptera arenaria* | SCBG983 | MK753796 | MK704819 | MK691890 | MK756558 | \\\ |
| 12 | *Allagoptera arenaria* | XMBG005 | MK753797 | MK704820 | MK691891 | MK756559 | \\\ |
| 13 | *Allagoptera caudescens* | SCBG038 | MK753798 | MK704821 | MK691892 | MK756547 | \\\ |
| 14 | *Allagoptera caudescens* | XTBG125 | MK753799 | MK704822 | MK691893 | MK756548 | \\\ |
| 15 | *Archontophoenix alexandrae* | SCBG887 | MK753683 | MK704878 | MK692161 | MK756826 | MK683292 |
| 16 | *Archontophoenix alexandrae* | XTBG038 | MK753684 | MK704879 | MK692162 | MK756827 | \\\ |
| 17 | *Archontophoenix cunninghamiana* | SCBG059 | MK753685 | MK704880 | MK692163 | MK756831 | \\\ |
| 18 | *Archontophoenix cunninghamiana* | XMBG078 | MK753686 | MK704881 | MK692164 | MK756832 | \\\ |
| 19 | *Archontophoenix purpurea* | XMBG032 | MK753687 | MK704882 | MK692173 | MK756828 | \\\ |
| 20 | *Archontophoenix purpurea* | XMBG061 | MK753688 | MK704883 | MK692174 | MK756829 | \\\ |
| 21 | *Archontophoenix purpurea* | XTBG237 | MK753689 | MK704884 | MK692175 | MK756830 | MK683293 |
| 22 | *Areca catechu* | SCBG943 | MK753921 | MK705056 | MK692306 | MK756922 | \\\ |
| 23 | *Areca catechu* | XMBG131 | MK753922 | MK705057 | MK692307 | MK756929 | \\\ |
| 24 | *Areca catechu* | XTBG097 | MK753923 | MK764362 | MK692308 | MK756927 | \\\ |
| 25 | *Areca catechu* | XTBG239 | MK753924 | MK764363 | MK692309 | MK756928 | \\\ |
| 26 | *Areca concinna* | XTBG062 | MK753939 | MK705060 | \\\ | MK756925 | \\\ |
| 27 | *Areca sp.* | SCBG125 | MK753940 | MK705058 | \\\ | MK756923 | \\\ |
| 28 | *Areca sp.* | SCBG998 | MK753947 | MK704951 | \\\ | MK756926 | \\\ |
| 29 | *Areca sp.* | XTBG099 | MK753938 | MK705061 | \\\ | MK756897 | \\\ |
| 30 | *Areca triandra* | SCBG873 | MK753941 | MK705059 | \\\ | MK756924 | \\\ |
| 31 | *Areca vestiaria* | XTBG096 | MK753942 | MK705054 | \\\ | MK756942 | \\\ |
| 32 | *Areca vestiaria* | XTBG098 | MK753925 | MK705053 | MK692310 | MK756906 | MK683332 |
| 33 | *Areca vestiaria* | XTBG101 | MK753943 | MK705055 | \\\ | MK756943 | MK683333 |
| 34 | *Arenga australasica* | XTBG083 | MK753492 | MK705109 | MK692438 | MK756683 | \\\ |
| 35 | *Arenga caryotoides* | XTBG059 | MK753528 | MK705138 | MK692440 | MK756727 | MK683429 |
| 36 | *Arenga caryotoides* | XTBG060 | MK753476 | MK705141 | MK692441 | MK756730 | MK683430 |
| 37 | *Arenga caudata* | SCBG010 | MK753505 | MK705112 | MK692453 | MK756684 | \\\ |
| 38 | *Arenga caudata* | SCBG870 | MK753508 | MK705115 | MK692456 | MK756689 | MK683434 |
| 39 | *Arenga caudata* | SCBG961 | MK753509 | MK705116 | MK692457 | MK756694 | MK683435 |
| 40 | *Arenga caudata* | SCBG966 | MK753510 | MK705117 | MK692458 | MK756690 | MK683436 |
| 41 | *Arenga caudata* | XMBG109 | MK753506 | MK705113 | MK692454 | MK756685 | \\\ |
| 42 | *Arenga caudata* | XTBG176 | MK753507 | MK705114 | MK692455 | MK756687 | MK683438 |
| 43 | *Arenga caudata* | XTBG179 | MK753511 | MK705118 | MK692459 | MK756691 | MK683437 |
| 44 | *Arenga caudata* | XTBG211 | MK753512 | MK705119 | MK692460 | MK756686 | MK683439 |
| 45 | *Arenga disticha* | XMBG022 | MK753515 | MK705144 | MK692464 | MK756731 | MK683441 |
| 46 | *Arenga disticha* | XMBG054 | MK753516 | MK705145 | MK692465 | MK756732 | MK683442 |
| 47 | *Arenga disticha* | XMBG139 | MK753517 | MK705146 | MK692466 | MK756733 | MK683443 |
| 48 | *Arenga engleri* | SCBG085 | MK753498 | MK705147 | MK692447 | MK756699 | MK683421 |
| 49 | *Arenga engleri* | SCBG126 | MK753499 | MK705148 | MK692448 | MK756700 | MK683422 |
| 50 | *Arenga gracilis* | SCBG016 | MK753496 | MK705139 | MK692444 | MK756728 | MK683432 |
| 51 | *Arenga hookeriana* | XMBG401 | MK753513 | MK705120 | MK692461 | MK756692 | MK683440 |
| 52 | *Arenga oblongifolia* | SCBG007 | MK753494 | MK705142 | MK692442 | MK756734 | \\\ |
| 53 | *Arenga oblongifolia* | XMBG073 | MK753495 | MK705143 | MK692443 | MK756735 | MK683431 |
| 54 | *Arenga obtusifolia* | XTBG084 | MK753504 | MK705111 | MK692446 | MK756688 | MK683427 |
| 55 | *Arenga pinnata* | SCBG002 | MK753529 | MK705121 | MK692462 | MK756696 | \\\ |
| 56 | *Arenga ryukyuensis* | SCBG151 | MK753500 | MK705149 | MK692449 | MK756701 | MK683423 |
| 57 | *Arenga ryukyuensis* | XMBG135 | MK753501 | MK705150 | MK692450 | MK756702 | MK683426 |
| 58 | *Arenga ryukyuensis* | XMBG209 | MK753502 | MK705151 | MK692451 | MK756703 | MK683424 |
| 59 | *Arenga sp.* | XMBG229 | MK753503 | MK705152 | MK692452 | MK756704 | MK683425 |
| 60 | *Arenga sp.* | XTBG221 | MK753497 | MK705140 | MK692445 | MK756729 | MK683433 |
| 61 | *Arenga tremula* | XTBG029 | MK753493 | MK705110 | MK692439 | MK756998 | \\\ |
| 62 | *Arenga westerhoutii* | SCBG897 | MK753514 | MK705122 | MK692463 | MK756697 | MK683428 |
| 63 | *Astrocaryum alatum* | SCBG021 | MK753772 | MK704766 | MK691803 | MK756680 | MK683194 |
| 64 | *Astrocaryum alatum* | SCBG079 | MK753765 | MK704761 | MK691804 | MK756681 | \\\ |
| 65 | *Astrocaryum alatum* | XMBG040 | MK753766 | MK704762 | MK691805 | MK756682 | MK683195 |
| 66 | *Astrocaryum murumuru* | SCBG073 | MK753396 | MK704865 | MK691799 | MK756812 | MK683191 |
| 67 | *Astrocaryum murumuru* | XMBG015 | MK753397 | MK704866 | MK691800 | MK756813 | MK683192 |
| 68 | *Astrocaryum standleyanum* | XTBG013 | MK753398 | MK704763 | MK691801 | MK756677 | MK683193 |
| 69 | *Attalea amygdalina* | SCBG899 | MK753830 | MK704850 | MK691828 | MK756373 | MK683113 |
| 70 | *Attalea amygdalina* | SCBG948 | MK753831 | MK704851 | MK691829 | MK756374 | \\\ |
| 71 | *Attalea amygdalina* | XTBG003 | MK753832 | MK704852 | MK691830 | MK756375 | \\\ |
| 72 | *Attalea butyracea* | XTBG161 | MK753833 | MK704853 | MK691827 | MK756376 | MK683115 |
| 73 | *Attalea cohune* | SCBG946 | MK753834 | MK704854 | MK691831 | MK756377 | \\\ |
| 74 | *Attalea cohune* | XMBG174 | MK753835 | MK704855 | MK691832 | MK756378 | MK683114 |
| 75 | *Attalea maripa* | XTBG178 | MK753826 | MK704846 | MK691894 | MK756384 | MK683117 |
| 76 | *Attalea oleifera* | SCBG084 | MK753836 | MK704856 | MK691833 | MK756380 | MK683116 |
| 77 | *Attalea oleifera* | XMBG232 | MK753837 | MK704857 | MK691834 | MK756381 | MK683118 |
| 78 | *Attalea oleifera* | XTBG159 | MK753838 | MK704858 | MK691835 | MK756382 | \\\ |
| 79 | *Attalea oleifera* | XTBG160 | MK753839 | MK704859 | MK691836 | MK756383 | \\\ |
| 80 | *Attalea phalerata* | XMBG036 | MK753827 | MK704847 | MK691895 | MK756385 | \\\ |
| 81 | *Attalea phalerata* | XMBG076 | MK753828 | MK704848 | MK691896 | MK757066 | \\\ |
| 82 | *Attalea rostrata* | XTBG001 | MK753829 | MK704849 | MK691897 | MK756379 | \\\ |
| 83 | *Bactris gasipaes* | SCBG018 | MK753895 | MK704774 | \\\ | MK756785 | MK683203 |
| 84 | *Bactris gasipaes* | SCBG981 | MK753889 | MK704768 | MK691817 | MK756782 | \\\ |
| 85 | *Bactris gasipaes* | XTBG009 | MK753890 | MK704769 | MK691818 | MK756783 | MK683209 |
| 86 | *Bactris gasipaes* | XTBG012 | MK753891 | MK704770 | MK691819 | MK756784 | \\\ |
| 87 | *Bactris major* | SCBG013 | MK753775 | MK704842 | MK691823 | MK756666 | MK683210 |
| 88 | *Bactris major* | SCBG049 | MK753776 | MK704843 | MK691824 | MK756667 | MK683206 |
| 89 | *Bactris major* | XMBG119 | MK753777 | MK704844 | MK691825 | MK756668 | MK683207 |
| 90 | *Bactris mexicana* | XMBG309 | MK753894 | MK704773 | MK691822 | MK756665 | MK683208 |
| 91 | *Bactris setulosa* | XMBG117 | MK753892 | MK704771 | MK691820 | MK756786 | MK683204 |
| 92 | *Bactris sp.* | XMBG422 | MK753893 | MK704772 | MK691821 | MK756787 | MK683205 |
| 93 | *Balaka seemannii* | XTBG053 | MK753679 | MK704948 | MK692226 | MK756961 | MK683289 |
| 94 | *Balaka seemannii* | XTBG191 | MK753680 | MK704949 | MK692227 | MK756962 | MK683290 |
| 95 | *Balaka seemannii* | XTBG207 | MK753681 | MK704950 | MK692228 | MK756963 | MK683291 |
| 96 | *Beccariophoenix alfredii* | XMBG236 | MK753841 | MK704833 | MK691838 | MK756393 | \\\ |
| 97 | *Beccariophoenix alfredii* | XMBG239 | MK753842 | MK704834 | MK691839 | MK756411 | \\\ |
| 98 | *Beccariophoenix fenestralis* | SCBG098 | MK753843 | MK704824 | MK691843 | MK756483 | MK683123 |
| 99 | *Beccariophoenix madagascariensis* | SCBG086 | MK753849 | MK704828 | \\\ | MK756487 | \\\ |
| 100 | *Beccariophoenix madagascariensis* | SCBG997 | MK753847 | MK704826 | MK691844 | MK756485 | MK683127 |
| 101 | *Beccariophoenix madagascariensis* | XMBG010 | MK753848 | MK704827 | MK691845 | MK756486 | MK683128 |
| 102 | *Beccariophoenix madagascariensis* | XMBG033 | MK753850 | MK704829 | \\\ | MK756488 | MK683129 |
| 103 | *Beccariophoenix sp.* | SCBG113 | MK753845 | MK704830 | MK691841 | MK756489 | MK683124 |
| 104 | *Beccariophoenix sp.* | SCBG130 | MK753844 | MK704825 | MK691840 | MK756484 | MK683125 |
| 105 | *Beccariophoenix sp.* | XTBG049 | MK753846 | MK704831 | MK691842 | MK756490 | MK683126 |
| 106 | *Bentinckia nicobarica* | XTBG227 | MK753948 | MK704867 | \\\ | MK756957 | \\\ |
| 107 | *Bismarckia nobilis* | SCBG894 | MK753402 | MK705078 | MK692338 | MK756794 | MK683218 |
| 108 | *Bismarckia nobilis* | XMBG408 | MK753403 | MK705079 | MK692339 | MK756795 | \\\ |
| 109 | *Borassodendron machadonis* | SCBG104 | MK753410 | MK705095 | MK692348 | MK756992 | \\\ |
| 110 | *Borassodendron machadonis* | XTBG018 | MK753411 | MK705096 | MK692349 | MK756993 | MK683226 |
| 111 | *Borassus aethiopum* | SCBG849 | MK753412 | MK705084 | MK692350 | MK756800 | MK683227 |
| 112 | *Borassus flabellifer* | SCBG015 | MK753414 | MK705086 | MK692352 | MK756804 | \\\ |
| 113 | *Borassus flabellifer* | XMBG023 | MK753415 | MK705087 | MK692353 | MK756805 | \\\ |
| 114 | *Borassus flabellifer* | XMBG241 | MK753416 | MK705088 | MK692354 | MK756806 | \\\ |
| 115 | *Borassus madagascariensis* | SCBG173 | MK753417 | MK705089 | MK692355 | MK756801 | MK683228 |
| 116 | *Borassus madagascariensis* | XTBG058 | MK753418 | MK705090 | MK692356 | MK756802 | MK683229 |
| 117 | *Borassus aethiopum* | XTBG174 | MK753413 | MK705085 | MK692351 | MK756803 | MK683230 |
| 118 | *Brahea aculeata* | XMBG411 | MK753546 | MK704520 | MK691902 | MK756327 | \\\ |
| 119 | *Brahea armata* | XTBG193 | MK753547 | MK704571 | MK691905 | MK756329 | \\\ |
| 120 | *Brahea brandegeei* | XMBG125 | MK753548 | MK704581 | MK691904 | MK756328 | \\\ |
| 121 | *Brahea dulcis* | XMBG198 | MK753541 | MK704554 | MK691911 | MK756344 | MK683151 |
| 122 | *Brahea sarukhanii* | XMBG197 | MK753542 | MK704552 | MK691909 | MK756332 | MK683147 |
| 123 | *Brassiophoenix drymophloeoides* | XTBG118 | MK753682 | MK704942 | MK692229 | MK756867 | MK683285 |
| 124 | *Burretiokentia hapala* | SCBG089 | MK753698 | MK704895 | MK692237 | MK756930 | MK683296 |
| 125 | *Burretiokentia hapala* | SCBG195 | MK753699 | MK704896 | MK692238 | MK756931 | MK683297 |
| 126 | *Burretiokentia hapala* | XMBG185 | MK753700 | MK704897 | MK692239 | MK756932 | MK683299 |
| 127 | *Burretiokentia hapala* | XMBG223 | MK753701 | MK704898 | MK692240 | MK756933 | MK683300 |
| 128 | *Burretiokentia hapala* | XMBG320 | MK753702 | MK704899 | MK692241 | MK756934 | MK683298 |
| 129 | *Burretiokentia hapala* | XMBG438 | MK753703 | MK704900 | MK692242 | MK756935 | \\\ |
| 130 | *Butia capitata* | SCBG875 | MK753954 | MK704813 | MK691854 | MK756394 | \\\ |
| 131 | *Butia eriospatha* | XMBG002 | MK753957 | MK704816 | MK691857 | MK756397 | \\\ |
| 132 | *Butia capitata* | XMBG068 | MK753955 | MK704814 | MK691855 | MK756395 | \\\ |
| 133 | *Butia capitata* | SCBG149 | MK753956 | MK704815 | MK691856 | MK756396 | \\\ |
| 134 | *Butia eriospatha* | XMBG416 | MK753958 | MK704817 | MK691858 | MK756398 | MK683112 |
| 135 | *Calamus acanthospathus* | XTBG146 | MK753994 | MK705191 | MK692376 | MK757010 | MK683374 |
| 136 | *Calamus curranii* | XTBG081 | MK754041 | MK705198 | MK692401 | MK757014 | MK683392 |
| 137 | *Calamus erectus* | XTBG076 | MK754001 | MK705195 | MK692405 | MK757024 | MK683407 |
| 138 | *Calamus erectus* | XTBG143 | MK754002 | MK705196 | MK692406 | MK757025 | MK683394 |
| 139 | *Calamus exilis* | XTBG132 | MK754054 | MK705234 | MK692413 | MK757018 | \\\ |
| 140 | *Calamus exilis* | XTBG141 | MK754055 | MK705235 | MK692414 | MK757019 | \\\ |
| 141 | *Calamus flagellum* | XMBG421 | MK754003 | MK705202 | MK692407 | MK757026 | MK683395 |
| 142 | *Calamus flagellum* | XTBG149 | MK754004 | MK705203 | MK692408 | MK757027 | MK683396 |
| 143 | *Calamus floribundus* | XTBG155 | MK754006 | MK705231 | MK692410 | MK757011 | MK683376 |
| 144 | *Calamus guruba* | XTBG069 | MK753995 | MK705226 | MK692412 | MK757050 | MK683401 |
| 145 | *Calamus henryanus* | XTBG080 | MK753997 | MK705192 | MK692382 | MK757030 | MK683405 |
| 146 | *Calamus javensis* | XTBG133 | MK754038 | MK705232 | MK692380 | MK757012 | \\\ |
| 147 | *Calamus javensis* | XTBG185 | MK754039 | MK705233 | MK692381 | MK757013 | MK683375 |
| 148 | *Calamus jenkinsianus* | XTBG071 | MK753998 | MK705199 | MK692402 | MK757015 | MK683408 |
| 149 | *Calamus jenkinsianus* | XTBG142 | MK753999 | MK705200 | MK692403 | MK757016 | MK683409 |
| 150 | *Calamus jenkinsianus* | XTBG228 | MK754000 | MK705201 | MK692404 | MK757017 | MK683393 |
| 151 | *Calamus inermis* | XTBG078 | MK754015 | MK705210 | MK692383 | MK757035 | MK683378 |
| 152 | *Calamus inermis* | XTBG131 | MK754016 | MK705211 | MK692384 | MK757036 | MK683383 |
| 153 | *Calamus inermis* | XTBG135 | MK754017 | MK705212 | MK692385 | MK757037 | MK683380 |
| 154 | *Calamus inermis* | XTBG140 | MK754018 | MK705213 | MK692386 | MK757038 | MK683384 |
| 155 | *Calamus inermis* | XTBG147 | MK754019 | MK705214 | MK692387 | MK757039 | MK683382 |
| 156 | *Calamus inermis* | XTBG182 | MK754020 | MK705215 | MK692388 | MK757040 | MK683385 |
| 157 | *Calamus inermis* | XTBG184 | MK754021 | MK705216 | MK692389 | MK757041 | MK683381 |
| 158 | *Calamus latifolius* | XTBG072 | MK754026 | MK705217 | MK692390 | MK757047 | MK683379 |
| 159 | *Calamus latifolius* | XTBG137 | MK754027 | MK705218 | MK692391 | MK757048 | \\\ |
| 160 | *Calamus simplicifolius* | XMBG096 | MK754028 | MK705219 | MK692393 | MK757042 | MK683387 |
| 161 | *Calamus simplicifolius* | XMBG437 | MK754029 | MK705220 | MK692394 | MK757043 | \\\ |
| 162 | *Calamus sp.* | SCBG107 | MK754030 | MK705204 | MK692415 | MK757055 | \\\ |
| 163 | *Calamus sp.* | SCBG110 | MK754022 | MK705221 | MK692395 | MK757044 | MK683400 |
| 164 | *Calamus sp.* | SCBG124 | MK754031 | MK705205 | MK692416 | MK757056 | \\\ |
| 165 | *Calamus sp.* | SCBG129 | MK754005 | MK705197 | MK692409 | MK757028 | \\\ |
| 166 | *Calamus sp.* | XMBG434 | MK754023 | MK705222 | MK692396 | MK757045 | MK683388 |
| 167 | *Calamus sp.* | XTBG130 | MK754025 | MK705227 | MK692392 | MK757049 | MK683386 |
| 168 | *Calamus sp.* | XTBG145 | MK754024 | MK705223 | MK692397 | MK757046 | MK683389 |
| 169 | *Calamus sp.* | XTBG183 | MK754036 | MK705225 | MK692411 | MK757029 | MK683391 |
| 170 | *Calamus tetradactylus* | XTBG136 | MK754014 | MK705224 | MK692398 | MK757031 | MK683390 |
| 171 | *Calamus thysanolepis* | XMBG013 | MK754032 | MK705206 | MK692417 | MK757020 | MK683402 |
| 172 | *Calamus thysanolepis* | XMBG014 | MK754033 | MK705207 | MK692418 | MK757021 | MK683403 |
| 173 | *Calamus thysanolepis* | XMBG433 | MK754034 | MK705208 | MK692419 | MK757022 | MK683399 |
| 174 | *Calamus thysanolepis* | XTBG152 | MK754035 | MK705209 | MK692420 | MK757023 | MK683404 |
| 175 | *Calamus viminalis* | SCBG134 | MK754011 | MK705228 | MK692377 | MK757032 | \\\ |
| 176 | *Calamus viminalis* | XTBG079 | MK754012 | MK705229 | MK692378 | MK757033 | MK683406 |
| 177 | *Calamus viminalis* | XTBG139 | MK754013 | MK705230 | MK692379 | MK757034 | MK683377 |
| 178 | *Calamus walkeri* | XTBG134 | MK754009 | MK705193 | MK692399 | MK757053 | MK683397 |
| 179 | *Calamus walkeri* | XTBG156 | MK754010 | MK705194 | MK692400 | MK757054 | MK683398 |
| 180 | *Calyptrocalyx albertisianus* | XTBG092 | MK753743 | MK704911 | \\\ | MK756371 | \\\ |
| 181 | *Calyptrocalyx elegans* | XTBG093 | MK753707 | MK704962 | MK692222 | MK756351 | MK683302 |
| 182 | *Calyptrocalyx elegans* | XTBG119 | MK753744 | MK704965 | MK692225 | \\\ | MK683331 |
| 183 | *Calyptrocalyx forbesii* | XTBG090 | MK753708 | MK704963 | MK692223 | MK756352 | MK683303 |
| 184 | *Calyptrocalyx forbesii* | XTBG091 | MK753709 | MK704964 | MK692224 | MK756353 | MK683304 |
| 185 | *Carpentaria acuminata* | SCBG070 | MK753636 | MK704952 | MK692209 | MK756856 | MK683277 |
| 186 | *Carpentaria acuminata* | SCBG099 | MK753637 | MK704953 | MK692210 | MK756857 | MK683278 |
| 187 | *Carpentaria acuminata* | SCBG167 | MK753638 | MK704954 | MK692211 | MK756858 | MK683279 |
| 188 | *Carpentaria acuminata* | XMBG016 | MK753639 | MK704955 | MK692212 | MK756859 | MK683280 |
| 189 | *Carpentaria acuminata* | XMBG042 | MK753640 | MK704956 | MK692213 | MK756860 | MK683281 |
| 190 | *Carpentaria acuminata* | XMBG111 | MK753641 | MK704957 | MK692214 | MK756861 | MK683284 |
| 191 | *Carpentaria acuminata* | XMBG179 | MK753642 | MK704958 | MK692215 | MK756862 | MK683282 |
| 192 | *Carpentaria acuminata* | XMBG221 | MK753643 | MK704959 | MK692216 | MK756863 | MK683283 |
| 193 | *Carpoxylon macrospermum* | XTBG052 | MK753710 | MK704919 | MK692252 | MK756807 | \\\ |
| 194 | *Carpoxylon macrospermum* | XTBG195 | MK753711 | MK704920 | MK692254 | MK756808 | \\\ |
| 195 | *Carpoxylon macrospermum* | XTBG234 | MK753712 | MK705000 | MK692253 | MK756809 | \\\ |
| 196 | *Caryota cumingii* | XMBG018 | MK753530 | MK705123 | MK692467 | MK756713 | MK683444 |
| 197 | *Caryota maxima* | SCBG896 | MK753520 | MK705133 | MK692471 | MK756724 | MK683447 |
| 198 | *Caryota maxima* | XMBG021 | MK753521 | MK705134 | MK692472 | MK756725 | MK683448 |
| 199 | *Caryota maxima* | XMBG405 | MK753522 | MK705135 | MK692473 | MK756726 | MK683449 |
| 200 | *Caryota mitis* | SCBG892 | MK753526 | MK705130 | \\\ | MK756718 | \\\ |
| 201 | *Caryota mitis* | XTBG066 | MK753519 | MK705129 | MK692470 | MK756717 | MK683445 |
| 202 | *Caryota monostachya* | SCBG128 | MK753527 | MK705125 | \\\ | MK756715 | \\\ |
| 203 | *Caryota monostachya* | XTBG064 | MK753518 | MK705124 | MK692468 | MK756714 | \\\ |
| 204 | *Caryota obtusa* | SCBG872 | MK753523 | MK705136 | MK692474 | MK756721 | \\\ |
| 205 | *Caryota obtusa* | XMBG087 | MK753524 | MK705137 | MK692475 | MK756722 | \\\ |
| 206 | *Caryota sp.* | XMBG019 | MK753532 | MK705126 | \\\ | MK756719 | \\\ |
| 207 | *Caryota sp.* | XMBG020 | MK753533 | MK705127 | \\\ | MK756720 | \\\ |
| 208 | *Caryota sympetala* | SCBG980 | MK753534 | MK705131 | \\\ | MK756716 | \\\ |
| 209 | *Caryota urens* | SCBG062 | MK753531 | MK705128 | MK692469 | MK756723 | MK683446 |
| 210 | *Caryota zebrina* | SCBG153 | MK753525 | MK705132 | MK692476 | MK756695 | MK683450 |
| 211 | *Chamaedorea anemophila* | SCBG876 | MK753897 | MK764364 | MK692479 | MK756638 | MK683356 |
| 212 | *Chamaedorea anemophila* | SCBG986 | MK753898 | MK764365 | MK692480 | MK756639 | MK683357 |
| 213 | *Chamaedorea cataractarum* | SCBG019 | MK753902 | MK705172 | MK692487 | MK756660 | \\\ |
| 214 | *Chamaedorea costaricana* | XMBG044 | MK753914 | MK705181 | MK692492 | MK756655 | MK683365 |
| 215 | *Chamaedorea costaricana* | XMBG430 | MK753915 | MK705182 | MK692493 | MK756656 | MK683366 |
| 216 | *Chamaedorea costaricana* | XTBG199 | MK753916 | MK705183 | MK692494 | MK756657 | MK683367 |
| 217 | *Chamaedorea elegans* | SCBG863 | MK753917 | MK705178 | MK692501 | MK756650 | MK683364 |
| 218 | *Chamaedorea ernesti-augusti* | SCBG095 | MK753906 | MK705175 | MK692490 | MK756651 | MK683361 |
| 219 | *Chamaedorea glaucifolia* | SCBG071 | MK753901 | MK705166 | MK692483 | MK756640 | \\\ |
| 220 | *Chamaedorea hooperiana* | XMBG193 | MK753899 | MK705163 | MK692481 | MK756641 | MK683358 |
| 221 | *Chamaedorea hooperiana* | XMBG196 | MK753900 | MK705164 | MK692482 | MK756642 | \\\ |
| 222 | *Chamaedorea hooperiana* | XTBG202 | MK753913 | MK705165 | \\\ | MK756643 | \\\ |
| 223 | *Chamaedorea linearis* | XMBG431 | MK753905 | MK705184 | MK692486 | MK756654 | \\\ |
| 224 | *Chamaedorea metallica* | SCBG068 | MK753907 | MK705177 | MK692491 | MK756652 | \\\ |
| 225 | *Chamaedorea radicalis* | XMBG011 | MK753918 | MK705173 | MK692484 | MK756658 | MK683368 |
| 226 | *Chamaedorea radicalis* | XMBG035 | MK753919 | MK705174 | MK692485 | MK756659 | MK683369 |
| 227 | *Chamaedorea seifrizii* | SCBG879 | MK753911 | MK705179 | MK692498 | MK756661 | MK683370 |
| 228 | *Chamaedorea seifrizii* | XTBG030 | MK753912 | MK705180 | MK692499 | MK756662 | MK683371 |
| 229 | *Chamaedorea stolonifera* | XMBG403 | MK753908 | MK705176 | MK692495 | MK756653 | MK683362 |
| 230 | *Chamaedorea tepejilote* | SCBG001 | MK753903 | MK705171 | MK692489 | MK756649 | MK683372 |
| 231 | *Chamaedorea tepejilote* | XMBG138 | MK753904 | MK705162 | MK692488 | MK756648 | \\\ |
| 232 | *Chamaedorea warscewiczii* | XMBG207 | MK753909 | MK705169 | MK692496 | MK756646 | MK683359 |
| 233 | *Chamaedorea warscewiczii* | XMBG318 | MK753910 | MK705170 | MK692497 | MK756647 | MK683360 |
| 234 | *Chamaerops humilis* | XMBG012 | MK753557 | MK704611 | MK692113 | MK756492 | \\\ |
| 235 | *Chamaerops humilis* | XMBG060 | MK753558 | MK704612 | MK692114 | MK756493 | MK683159 |
| 236 | *Chamaerops humilis* | XMBG137 | MK753559 | MK704613 | MK692115 | MK756494 | MK683160 |
| 237 | *Chamaerops humilis var. argentea* | XMBG059 | MK753560 | MK704614 | MK692116 | MK756495 | MK683161 |
| 238 | *Chambeyronia macrocarpa* | SCBG074 | MK753690 | MK704891 | MK692165 | MK756833 | MK674498 |
| 239 | *Chambeyronia macrocarpa* | SCBG169 | MK753691 | MK704892 | MK692166 | MK756851 | MK674501 |
| 240 | *Chambeyronia macrocarpa* | XMBG067 | MK753692 | MK704893 | MK692167 | MK756852 | MK674499 |
| 241 | *Chuniophoenix hainanensis* | SCBG987 | MK753881 | MK705103 | MK692362 | MK756994 | \\\ |
| 242 | *Chuniophoenix nana* | SCBG127 | MK753888 | MK705104 | MK692363 | MK756995 | \\\ |
| 243 | *Chuniophoenix nana* | SCBG963 | MK753882 | MK705105 | MK692364 | MK756996 | \\\ |
| 244 | *Coccothrinax argentata* | SCBG984 | MK753584 | MK704689 | MK692015 | MK756599 | \\\ |
| 245 | *Coccothrinax argentata* | XMBG086 | MK753585 | MK704690 | MK692016 | MK756602 | MK683079 |
| 246 | *Coccothrinax barbadensis* | XTBG214 | MK753591 | MK704681 | MK692022 | MK756606 | MK683092 |
| 247 | *Coccothrinax crinita* | XMBG402 | MK753586 | MK704691 | MK692017 | MK756604 | MK683084 |
| 248 | *Coccothrinax miraguama* | SCBG995 | MK753587 | MK704692 | MK692018 | MK756605 | MK683080 |
| 249 | *Coccothrinax miraguama* | XMBG120 | MK753562 | MK704582 | \\\ | MK756518 | \\\ |
| 250 | *Coccothrinax miraguama* | XMBG199 | MK753588 | MK704693 | MK692019 | MK756603 | \\\ |
| 251 | *Coccothrinax proctorii* | XTBG217 | MK753597 | MK704697 | MK692030 | MK756607 | MK683089 |
| 252 | *Coccothrinax readii* | SCBG170 | MK753598 | MK704698 | MK692031 | MK756608 | MK683082 |
| 253 | *Coccothrinax sp.* | SCBG097 | MK753589 | MK704694 | MK692020 | MK756600 | MK683081 |
| 254 | *Coccothrinax sp.* | SCBG115 | MK753590 | MK704695 | MK692021 | MK756601 | \\\ |
| 255 | *Coccothrinax sp.* | XMBG046 | MK753595 | MK704699 | MK692026 | MK756598 | MK683083 |
| 256 | *Coccothrinax sp.* | XMBG176 | MK753592 | MK704682 | MK692023 | MK756596 | MK683094 |
| 257 | *Coccothrinax sp.* | XMBG243 | MK753596 | MK704685 | MK692029 | MK756634 | \\\ |
| 258 | *Coccothrinax sp.* | XTBG187 | MK753608 | MK704696 | MK692027 | MK756609 | MK683090 |
| 259 | *Coccothrinax sp.* | XTBG197 | MK753593 | MK704683 | MK692024 | MK757067 | \\\ |
| 260 | *Coccothrinax spissa* | XTBG186 | MK753594 | MK704684 | MK692025 | MK756597 | MK683093 |
| 261 | *Cocos nucifera* | XMBG116 | MK753840 | MK704818 | MK691899 | MK756409 | \\\ |
| 262 | *Copernicia alba* | XMBG070 | MK753469 | MK704628 | MK691987 | MK756499 | \\\ |
| 263 | *Copernicia alba* | XMBG251 | MK753471 | MK704630 | MK691989 | MK756500 | \\\ |
| 264 | *Copernicia baileyana* | XMBG027 | MK753445 | MK704631 | MK691990 | MK756502 | MK683182 |
| 265 | *Copernicia baileyana* | XMBG028 | MK753472 | MK704632 | MK691991 | MK756503 | MK683183 |
| 266 | *Copernicia baileyana* | XMBG065 | MK753446 | MK704638 | MK691993 | MK756504 | \\\ |
| 267 | *Copernicia baileyana* | XMBG439 | MK753473 | MK704633 | MK691992 | MK756505 | \\\ |
| 268 | *Copernicia baileyana* | XTBG122 | MK753447 | MK704634 | MK691994 | MK756506 | \\\ |
| 269 | *Copernicia berteroana* | SCBG037 | MK753451 | MK704639 | MK691998 | MK756510 | MK683184 |
| 270 | *Copernicia glabrescens* | XTBG165 | MK753448 | MK704635 | MK691995 | MK756509 | MK683188 |
| 271 | *Copernicia hospita* | XTBG164 | MK753449 | MK704636 | MK691996 | MK756507 | MK674500 |
| 272 | *Copernicia macroglossa* | XMBG026 | MK753450 | MK704637 | MK691997 | MK756508 | MK683185 |
| 273 | *Copernicia prunifera* | SCBG992 | MK753470 | MK704629 | MK691988 | MK756501 | MK683189 |
| 274 | *Corypha umbraculifera* | SCBG121 | MK753392 | MK705153 | MK692357 | MK756969 | MK683222 |
| 275 | *Corypha umbraculifera* | XMBG114 | MK753393 | MK705154 | MK692358 | MK756970 | MK683223 |
| 276 | *Corypha utan* | SCBG985 | MK753399 | MK705155 | MK692359 | MK756971 | \\\ |
| 277 | *Corypha utan* | XMBG115 | MK753401 | MK705157 | MK692361 | MK756973 | MK683224 |
| 278 | *Corypha utan* | XTBG213 | MK753400 | MK705156 | MK692360 | MK756972 | MK683225 |
| 279 | *Cryosophila guagara* | XMBG074 | MK753857 | MK704706 | MK692061 | MK756622 | \\\ |
| 280 | *Cryosophila guagara* | XMBG075 | MK753858 | MK704707 | MK692062 | MK756623 | \\\ |
| 281 | *Cryosophila guagara* | XMBG080 | MK753859 | MK704708 | MK692063 | MK756624 | \\\ |
| 282 | *Cryosophila guagara* | XTBG020 | MK753860 | MK704709 | MK692064 | MK756625 | MK683181 |
| 283 | *Cryosophila warscewiczii* | SCBG953 | MK753861 | MK704710 | MK692065 | MK756613 | \\\ |
| 284 | *Cryosophila warscewiczii* | XMBG069 | MK753862 | MK704711 | MK692066 | MK756615 | \\\ |
| 285 | *Cryosophila warscewiczii* | XMBG081 | MK753863 | MK704712 | MK692067 | MK756614 | \\\ |
| 286 | *Cyphophoenix alba* | SCBG011 | MK753745 | MK704912 | MK692246 | \\\ | MK683305 |
| 287 | *Cyphophoenix elegans* | XMBG141 | MK753704 | MK704901 | MK692244 | MK756936 | MK683306 |
| 288 | *Cyphophoenix nucele* | XMBG177 | MK753705 | MK704902 | MK692245 | MK756937 | \\\ |
| 289 | *Cyrtostachys renda* | XTBG100 | MK753926 | MK705004 | MK692334 | MK756955 | \\\ |
| 290 | *Desmoncus orthacanthos* | XTBG022 | MK753773 | MK704764 | MK691806 | MK756678 | MK683196 |
| 291 | *Desmoncus orthacanthos* | XTBG151 | MK753774 | MK704765 | MK691807 | MK756679 | \\\ |
| 292 | *Dictyosperma album* | SCBG844 | MK753754 | MK704921 | \\\ | MK756877 | MK683334 |
| 293 | *Dictyosperma album* | XMBG098 | MK753755 | MK704922 | \\\ | MK756878 | MK683335 |
| 294 | *Dictyosperma album* | XMBG419 | MK753756 | MK704923 | \\\ | MK756879 | MK683337 |
| 295 | *Dictyosperma album var. aureum* | SCBG999 | MK753757 | MK704995 | \\\ | MK756880 | \\\ |
| 296 | *Dictyosperma album var. aureum* | XTBG019 | MK753758 | MK704924 | \\\ | MK756881 | MK683336 |
| 297 | *Dictyosperma album* | XTBG002 | MK753759 | MK704996 | \\\ | MK756882 | \\\ |
| 298 | *Dictyosperma sp.* | XMBG426 | MK753760 | MK704997 | \\\ | MK756883 | \\\ |
| 299 | *Ponapea hentyi* | XMBG410 | MK753644 | MK704960 | MK692186 | MK756959 | MK683255 |
| 300 | *Drymophloeus litigiosus* | XTBG189 | MK753645 | MK704961 | MK692187 | MK756960 | MK683256 |
| 301 | *Drymophloeus subdistichus* | XTBG233 | MK753746 | MK705068 | \\\ | MK756915 | \\\ |
| 302 | *Dypsis ankaizinensis* | SCBG132 | MK753713 | MK705001 | MK692277 | MK756892 | MK683233 |
| 303 | *Dypsis ankaizinensis* | SCBG168 | MK753714 | MK705002 | MK692278 | MK756893 | MK683234 |
| 304 | *Dypsis boiviniana* | SCBG094 | MK753734 | MK704936 | MK692286 | MK756823 | \\\ |
| 305 | *Dypsis cabadae* | SCBG028 | MK753715 | MK704904 | MK692275 | MK756543 | MK683237 |
| 306 | *Dypsis cabadae* | SCBG101 | MK753716 | MK704927 | MK692276 | MK756541 | MK683232 |
| 307 | *Dypsis carlsmithii* | SCBG122 | MK753721 | MK704932 | MK692262 | MK756552 | \\\ |
| 308 | *Dypsis carlsmithii* | SCBG139 | MK753722 | MK704933 | MK692263 | MK756553 | \\\ |
| 309 | *Dypsis decaryi* | SCBG888 | MK753749 | MK705008 | MK692285 | MK756890 | MK683240 |
| 310 | *Dypsis decaryi* | XMBG400 | MK753748 | MK705007 | MK692264 | MK756891 | MK683315 |
| 311 | *Dypsis decipiens* | SCBG078 | MK753726 | MK705011 | MK692267 | MK756545 | \\\ |
| 312 | *Dypsis decipiens* | SCBG111 | MK753728 | MK705013 | MK692270 | MK756546 | \\\ |
| 313 | *Dypsis decipiens* | XMBG099 | MK753727 | MK705012 | MK692268 | MK756941 | \\\ |
| 314 | *Dypsis lanceolata* | XTBG043 | MK753719 | MK705018 | MK692280 | MK756544 | \\\ |
| 315 | *Dypsis lanceolata* | XTBG044 | MK753720 | MK705015 | MK692281 | MK756894 | MK683314 |
| 316 | *Dypsis lastelliana* | XTBG004 | MK753732 | MK704930 | MK692282 | MK756549 | \\\ |
| 317 | *Dypsis leptocheilos* | SCBG880 | MK753733 | MK704931 | MK692283 | MK756896 | MK683241 |
| 318 | *Dypsis lutescens* | SCBG058 | MK753723 | MK704934 | MK692265 | MK756898 | MK683316 |
| 319 | *Dypsis lutescens* | SCBG146 | MK753724 | MK704935 | MK692266 | MK756899 | \\\ |
| 320 | *Dypsis madagascariensis* | SCBG090 | MK753718 | MK705003 | MK692279 | MK756895 | MK683235 |
| 321 | *Dypsis onilahensis* | SCBG161 | MK753729 | MK705009 | MK692269 | MK756888 | \\\ |
| 322 | *Dypsis onilahensis* | XMBG079 | MK753730 | MK705010 | MK692271 | MK756889 | \\\ |
| 323 | *Dypsis pembana* | XMBG034 | MK753717 | MK704928 | MK692274 | MK756542 | MK683236 |
| 324 | *Dypsis pinnatifrons* | SCBG045 | MK753735 | MK704938 | MK692287 | MK756900 | MK683242 |
| 325 | *Dypsis pinnatifrons* | XTBG225 | MK753736 | MK704939 | MK692288 | MK756938 | MK683243 |
| 326 | *Dypsis prestoniana* | XMBG230 | MK753725 | MK704905 | MK692260 | MK756887 | MK683239 |
| 327 | *Dypsis rivularis* | XMBG092 | MK753737 | MK704937 | MK692289 | MK756956 | \\\ |
| 328 | *Dypsis sp.* | SCBG140 | MK753731 | MK704929 | MK692284 | MK756940 | \\\ |
| 329 | *Dypsis utilis* | XMBG006 | MK753750 | MK704906 | MK692291 | MK756908 | \\\ |
| 330 | *Dypsis utilis* | XMBG097 | MK753751 | MK704907 | MK692292 | MK756909 | MK683321 |
| 331 | *Dypsis utilis* | XMBG231 | MK753752 | MK704908 | MK692293 | MK756910 | MK683322 |
| 332 | *Dypsis utilis* | XTBG094 | MK753753 | MK704909 | MK692294 | MK756911 | MK683323 |
| 333 | *Elaeis guineensis* | SCBG886 | MK753951 | MK704775 | MK692372 | MK756554 | MK683339 |
| 334 | *Elaeis guineensis* | XMBG037 | MK753952 | MK704776 | MK692373 | MK756555 | \\\ |
| 335 | *Elaeis guineensis* | XMBG124 | MK753953 | MK704767 | MK692374 | MK756556 | MK683340 |
| 336 | *Euterpe edulis* | XTBG192 | MK753767 | MK704971 | MK692300 | MK756817 | MK683328 |
| 337 | *Euterpe precatoria* | XTBG023 | MK753791 | MK704972 | MK692301 | MK756818 | MK683329 |
| 338 | *Euterpe precatoria* | XTBG027 | MK753792 | MK704973 | MK692302 | MK756819 | \\\ |
| 339 | *Euterpe precatoria* | XTBG230 | MK753793 | MK704974 | MK692303 | MK756820 | \\\ |
| 340 | *Euterpe precatoria* | XTBG232 | MK753794 | MK704975 | MK692304 | MK756821 | MK683330 |
| 341 | *Euterpe precatoria* | XTBG392 | MK753795 | MK705160 | \\\ | \\\ | \\\ |
| 342 | *Gaussia attenuata* | XTBG226 | MK753784 | MK704875 | MK691812 | MK756663 | MK683373 |
| 343 | *Gaussia maya* | XMBG045 | MK753785 | MK704876 | MK691813 | MK756664 | \\\ |
| 344 | *Geonoma longivaginata* | XTBG222 | MK753786 | MK704869 | MK692335 | MK756822 | \\\ |
| 345 | *Geonoma sp.* | XMBG412 | MK753768 | MK704868 | MK692305 | MK756824 | \\\ |
| 346 | *Guihaia grossifibrosa* | SCBG105 | MK753561 | MK704583 | MK692159 | MK756517 | \\\ |
| 347 | *Heterospathe elata* | SCBG069 | MK753870 | MK704903 | MK692247 | MK756550 | MK683317 |
| 348 | *Heterospathe elata* | XTBG218 | MK753871 | MK704917 | MK692248 | MK756551 | MK683318 |
| 349 | *Heterospathe elata* | XTBG231 | MK753872 | MK704918 | MK692249 | MK756871 | MK683319 |
| 350 | *Howea forsteriana* | XMBG091 | MK753738 | MK705024 | MK692231 | MK756868 | \\\ |
| 351 | *Howea forsteriana* | XMBG123 | MK753739 | MK705025 | MK692232 | MK756869 | \\\ |
| 352 | *Howea forsteriana* | XMBG194 | MK753740 | MK705026 | MK692233 | MK756870 | MK683307 |
| 353 | *Hydriastele microcarpa* | XTBG047 | MK753761 | MK704940 | MK692312 | MK756907 | \\\ |
| 354 | *Hydriastele wendlandiana* | XTBG054 | MK753762 | MK704910 | MK692313 | MK756901 | \\\ |
| 355 | *Hydriastele microspadix* | XTBG055 | MK753764 | MK705022 | \\\ | MK756903 | \\\ |
| 356 | *Hydriastele wendlandiana* | XTBG056 | MK753763 | MK704887 | MK692314 | MK756902 | \\\ |
| 357 | *Hyophorbe lagenicaulis* | SCBG093 | MK753787 | MK704861 | MK692503 | MK756561 | \\\ |
| 358 | *Hyophorbe sp.* | XMBG062 | MK753790 | MK704862 | \\\ | MK756562 | MK683176 |
| 359 | *Hyophorbe verschaffeltii* | XMBG030 | MK753788 | MK704863 | MK692504 | MK756563 | \\\ |
| 360 | *Hyophorbe verschaffeltii* | XMBG121 | MK753789 | MK704864 | MK692505 | MK756564 | \\\ |
| 361 | *Hyphaene coriacea* | XTBG120 | MK753404 | MK705094 | MK692341 | MK756780 | MK683215 |
| 362 | *Hyphaene thebaica* | XTBG121 | MK753405 | MK705093 | MK692343 | MK756781 | MK683216 |
| 363 | *Iguanura wallichiana* | XTBG089 | MK753741 | MK705034 | MK692299 | MK756991 | \\\ |
| 364 | *Johannesteijsmannia altifrons* | SCBG148 | MK753477 | MK704579 | MK691947 | MK756417 | \\\ |
| 365 | *Johannesteijsmannia altifrons* | XTBG017 | MK753478 | MK704580 | MK691948 | MK756418 | MK683175 |
| 366 | *Johannesteijsmannia magnifica* | XTBG016 | MK753479 | MK704577 | MK691946 | MK756416 | \\\ |
| 367 | *Jubaeopsis caffra* | XMBG093 | MK753769 | MK704836 | MK691898 | MK756412 | MK683119 |
| 368 | *Kentiopsis oliviformis* | XMBG083 | MK753694 | MK704913 | MK692169 | MK756945 | MK683310 |
| 369 | *Kentiopsis oliviformis* | XMBG122 | MK753695 | MK704914 | MK692170 | MK756946 | MK683311 |
| 370 | *Kentiopsis oliviformis* | XMBG420 | MK753696 | MK704915 | MK692171 | MK756947 | MK683294 |
| 371 | *Kentiopsis oliviformis* | XTBG087 | MK753697 | MK704916 | MK692172 | MK756948 | MK683312 |
| 372 | *Kentiopsis pyriformis* | XMBG303 | MK753693 | MK704894 | MK692168 | MK756874 | MK683295 |
| 373 | *Kerriodoxa elegans* | SCBG081 | MK753883 | MK705106 | MK692365 | MK756705 | MK683353 |
| 374 | *Kerriodoxa elegans* | XMBG077 | MK753884 | MK705107 | MK692366 | MK756706 | MK683354 |
| 375 | *Lanonia centralis* | SCBG020 | MK753463 | MK704561 | MK691980 | MK756477 | MK683154 |
| 376 | *Lanonia centralis* | SCBG877 | MK753464 | MK704562 | MK691981 | MK756478 | MK683155 |
| 377 | *Lanonia centralis* | XTBG025 | MK753465 | MK704563 | MK691982 | MK756479 | MK683156 |
| 378 | *Lanonia dasyantha* | SCBG890 | MK753466 | MK704564 | MK691983 | MK756480 | MK683157 |
| 379 | *Lanonia dasyantha* | XTBG031 | MK753467 | MK704565 | MK691984 | MK756481 | MK683158 |
| 380 | *Lanonia dasyantha* | XTBG105 | MK753468 | MK704566 | MK691985 | MK756482 | \\\ |
| 381 | *Latania loddigesii* | SCBG055 | MK753406 | MK705080 | MK692344 | MK756796 | MK683219 |
| 382 | *Latania loddigesii* | XMBG308 | MK753407 | MK705081 | MK692345 | MK756797 | MK683221 |
| 383 | *Latania lontaroides* | XTBG086 | MK753408 | MK705082 | MK692346 | MK756799 | MK683220 |
| 384 | *Latania verschaffeltii* | SCBG106 | MK753409 | MK705083 | MK692347 | MK756798 | \\\ |
| 385 | *Leucothrinax morrisii* | XMBG047 | MK753600 | MK704687 | MK692034 | MK756611 | MK683091 |
| 386 | *Leucothrinax morrisii* | XTBG188 | MK753601 | MK704688 | MK692035 | MK756612 | \\\ |
| 387 | *Licuala ferruginea* | XTBG110 | MK753991 | MK764369 | MK691974 | MK756431 | \\\ |
| 388 | *Licuala fordiana* | XMBG216 | MK753475 | \\\ | MK692117 | MK756433 | \\\ |
| 389 | *Licuala grandis* | XTBG108 | MK753535 | MK764370 | MK691972 | MK756496 | \\\ |
| 390 | *Licuala lauterbachii* | XTBG104 | MK753536 | MK764371 | MK691973 | MK756497 | \\\ |
| 391 | *Licuala paludosa* | XTBG106 | MK753992 | MK764377 | MK691977 | MK756424 | \\\ |
| 392 | *Licuala peltata* | SCBG912 | MK753474 | MK764381 | MK691971 | MK756434 | MK683137 |
| 393 | *Licuala ramsayi* | SCBG008 | MK753487 | MK764372 | MK691966 | MK756425 | \\\ |
| 394 | *Licuala ramsayi* | XMBG246 | MK753488 | MK764373 | MK691967 | MK756426 | MK683133 |
| 395 | *Licuala ramsayi* | XMBG406 | MK753489 | MK764374 | MK691968 | MK756427 | MK683134 |
| 396 | *Licuala ramsayi* | XTBG109 | MK753490 | MK764375 | MK691969 | MK756428 | \\\ |
| 397 | *Licuala ramsayi* | XTBG190 | MK753491 | MK764376 | MK691970 | MK756429 | MK683135 |
| 398 | *Licuala sp.* | XMBG066 | MK753993 | MK764378 | MK691978 | MK756430 | \\\ |
| 399 | *Linospadix monostachyos* | SCBG100 | MK753855 | MK705027 | MK692234 | MK756944 | \\\ |
| 400 | *Livistona alfredii* | XMBG003 | MK753419 | MK704525 | MK691918 | MK756442 | \\\ |
| 401 | *Livistona australis* | SCBG942 | MK753433 | MK704573 | MK691933 | MK756464 | MK726344 |
| 402 | *Livistona australis* | XMBG050 | MK753434 | MK704574 | MK691934 | MK756465 | MK726345 |
| 403 | *Livistona australis* | XMBG051 | MK753435 | MK704575 | MK691935 | MK756466 | MK726346 |
| 404 | *Livistona benthamii* | SCBG047 | MK753452 | MK704547 | MK692012 | MK756468 | MK683163 |
| 405 | *Livistona chinensis* | SCBG878 | MK753421 | MK704527 | MK691920 | MK756443 | MK683162 |
| 406 | *Livistona decora* | SCBG971 | MK753437 | MK704540 | MK691937 | MK756435 | \\\ |
| 407 | *Livistona decora* | XMBG024 | MK753438 | MK704541 | MK691938 | MK756436 | \\\ |
| 408 | *Livistona decora* | XMBG090 | MK753439 | MK704542 | MK691939 | MK756437 | \\\ |
| 409 | *Livistona decora* | XMBG132 | MK753440 | MK704543 | MK691940 | MK756438 | MK726351 |
| 410 | *Livistona drudei* | SCBG994 | MK753453 | MK704548 | MK692013 | MK756469 | MK726348 |
| 411 | *Livistona jenkinsiana* | SCBG882 | MK753422 | MK704528 | MK691921 | MK756444 | \\\ |
| 412 | *Livistona jenkinsiana* | XMBG053 | MK753423 | MK704529 | MK691922 | MK756445 | \\\ |
| 413 | *Livistona lanuginosa* | XTBG208 | MK753442 | MK704567 | MK691942 | MK756439 | MK683138 |
| 414 | *Livistona mariae* | XMBG248 | MK753441 | MK704544 | MK691941 | MK756440 | MK683139 |
| 415 | *Livistona muelleri* | SCBG064 | MK753459 | MK704521 | \\\ | MK756472 | MK683140 |
| 416 | *Livistona muelleri* | SCBG883 | MK753443 | MK704545 | MK691943 | MK756470 | MK726350 |
| 417 | *Livistona muelleri* | XMBG009 | MK753444 | MK704546 | MK691944 | MK756471 | MK683164 |
| 418 | *Livistona nitida* | XTBG114 | MK753436 | MK704539 | MK691936 | MK756441 | MK683141 |
| 419 | *Livistona saribus* | SCBG871 | MK753424 | MK704530 | MK691923 | MK756446 | MK683148 |
| 420 | *Livistona sp.* | SCBG048 | MK753460 | MK704522 | MK691932 | \\\ | \\\ |
| 421 | *Livistona sp.* | XMBG004 | MK753461 | MK704523 | \\\ | MK756473 | MK726349 |
| 422 | *Livistona sp.* | XMBG049 | MK753462 | MK704572 | \\\ | MK756467 | MK726347 |
| 423 | *Livistona sp.* | XMBG052 | MK753431 | MK704537 | MK691930 | MK756447 | MK726352 |
| 424 | *Livistona sp.* | XMBG301 | MK753432 | MK704538 | MK691931 | MK756448 | \\\ |
| 425 | *Livistona speciosa* | SCBG147 | MK753425 | MK704531 | MK691924 | MK756449 | \\\ |
| 426 | *Livistona speciosa* | SCBG811 | MK753426 | MK704532 | MK691925 | MK756450 | MK683149 |
| 427 | *Livistona sp.* | SCBG951 | MK753420 | MK704526 | MK691919 | MK756451 | \\\ |
| 428 | *Livistona speciosa* | SCBG989 | MK753427 | MK704533 | MK691926 | MK756455 | \\\ |
| 429 | *Livistona speciosa* | SCBG993 | MK753428 | MK704534 | MK691927 | MK756452 | MK726354 |
| 430 | *Livistona speciosa* | XMBG048 | MK753429 | MK704535 | MK691928 | MK756453 | \\\ |
| 431 | *Livistona speciosa* | XTBG117 | MK753430 | MK704536 | MK691929 | MK756454 | MK726353 |
| 432 | *Mauritia carana* | XTBG158 | MK754040 | MK705247 | MK692436 | MK757009 | \\\ |
| 433 | *Nannorrhops ritchieana* | XMBG085 | MK753887 | MK705102 | \\\ | MK756712 | \\\ |
| 434 | *Nannorrhops ritchieana* | XMBG254 | MK753885 | MK705099 | MK692369 | MK756709 | \\\ |
| 435 | *Nannorrhops ritchieana* | XTBG175 | MK753886 | MK705100 | MK692370 | MK756710 | MK683350 |
| 436 | *Nenga pumila var. pachystachya* | SCBG962 | MK753920 | MK705052 | MK692316 | MK756951 | \\\ |
| 437 | *Nephrosperma van-houtteanum* | XTBG103 | MK753747 | MK705032 | MK692296 | \\\ | \\\ |
| 438 | *Nephrosperma van-houtteanum* | XTBG181 | MK753742 | MK705031 | MK692295 | MK756990 | \\\ |
| 439 | *Normanbya normanbyi* | SCBG046 | MK753655 | MK704943 | MK692207 | MK756854 | MK683275 |
| 440 | *Normanbya normanbyi* | SCBG155 | MK753656 | MK704944 | MK692208 | MK756855 | MK683276 |
| 441 | *Oenocarpus bataua* | XTBG095 | MK753854 | MK704970 | MK692158 | MK756815 | \\\ |
| 442 | *Oraniopsis appendiculata* | SCBG133 | MK753633 | MK705020 | MK692119 | MK756737 | \\\ |
| 443 | *Oraniopsis appendiculata* | XMBG094 | MK753634 | MK705021 | MK692120 | MK756738 | MK683180 |
| 444 | *Pelagodoxa henryana* | XTBG238 | MK753864 | MK705033 | MK692336 | MK756816 | \\\ |
| 445 | *Phoenix acaulis* | XMBG084 | MK753959 | MK704670 | MK692088 | MK756772 | MK683073 |
| 446 | *Phoenix andamanensis* | SCBG112 | MK753967 | MK704654 | MK692094 | MK756759 | \\\ |
| 447 | *Phoenix andamanensis* | XMBG126 | MK753968 | MK704655 | MK692095 | MK756762 | \\\ |
| 448 | *Phoenix andamanensis* | XMBG127 | MK753969 | MK704656 | MK692096 | MK756763 | \\\ |
| 449 | *Phoenix andamanensis* | XTBG116 | MK753970 | MK704657 | MK692097 | MK756764 | MK683064 |
| 450 | *Phoenix atlantica* | SCBG041 | MK753986 | MK704664 | MK692109 | MK756755 | \\\ |
| 451 | *Phoenix atlantica* | SCBG054 | MK753987 | MK704665 | MK692110 | MK756756 | \\\ |
| 452 | *Phoenix canariensis* | XTBG115 | MK753989 | MK704678 | MK692112 | MK756765 | MK683070 |
| 453 | *Phoenix dactylifera* | SCBG056 | MK753974 | MK704658 | MK692104 | MK756760 | \\\ |
| 454 | *Phoenix dactylifera* | XMBG007 | MK753975 | MK704659 | MK692105 | MK756770 | \\\ |
| 455 | *Phoenix loureiroi* | SCBG103 | MK753960 | MK704671 | MK692089 | MK756773 | \\\ |
| 456 | *Phoenix loureiroi* | XMBG429 | MK753961 | MK704672 | MK692090 | MK756777 | MK683066 |
| 457 | *Phoenix paludosa* | SCBG123 | MK753962 | MK704673 | MK692091 | MK756774 | \\\ |
| 458 | *Phoenix paludosa* | SCBG967 | MK753963 | MK704674 | MK692092 | MK756776 | \\\ |
| 459 | *Phoenix paludosa* | XMBG129 | MK753964 | MK704675 | MK692093 | MK756775 | \\\ |
| 460 | *Phoenix pusilla* | SCBG065 | MK753965 | MK704676 | MK692102 | MK757001 | MK683067 |
| 461 | *Phoenix pusilla* | XTBG010 | MK753966 | MK704677 | MK692103 | MK757002 | MK683068 |
| 462 | *Phoenix reclinata* | SCBG042 | MK753978 | MK704662 | MK692107 | MK756771 | \\\ |
| 463 | *Phoenix reclinata* | SCBG057 | MK753979 | MK704663 | MK692108 | MK756757 | MK683071 |
| 464 | *Phoenix roebelenii* | SCBG061 | MK753980 | MK704648 | MK692086 | MK756748 | MK683060 |
| 465 | *Phoenix roebelenii* | SCBG874 | MK753982 | MK704650 | MK692082 | MK756749 | MK683061 |
| 466 | *Phoenix roebelenii* | XMBG089 | MK753983 | MK704651 | MK692083 | MK756750 | MK683063 |
| 467 | *Phoenix roebelenii* | XMBG227 | MK753984 | MK704652 | MK692084 | MK756751 | MK683076 |
| 468 | *Phoenix roebelenii* | XTBG007 | MK753985 | MK704653 | MK692085 | MK756752 | MK683062 |
| 469 | *Phoenix roebelenii* | XTBG008 | MK753981 | MK704649 | MK692087 | MK756753 | MK683074 |
| 470 | *Phoenix rupicola* | SCBG109 | MK753971 | MK704667 | MK692098 | MK756766 | \\\ |
| 471 | *Phoenix rupicola* | XMBG128 | MK753972 | MK704668 | MK692099 | MK756767 | \\\ |
| 472 | *Phoenix rupicola* | XTBG113 | MK753973 | MK704669 | MK692100 | MK756768 | MK683065 |
| 473 | *Phoenix sp.* | XMBG118 | MK753988 | MK704666 | MK692111 | MK756758 | MK683075 |
| 474 | *Phoenix sylvestris* | SCBG066 | MK753976 | MK704660 | MK692106 | MK756761 | MK683072 |
| 475 | *Phoenix theophrasti* | XTBG112 | MK753977 | MK704661 | MK692101 | MK756769 | MK683069 |
| 476 | *Phytelephas aequatorialis* | XTBG173 | MK753390 | MK705075 | MK692129 | MK756569 | \\\ |
| 477 | *Phytelephas macrocarpa* | XTBG172 | MK753391 | MK705076 | MK692130 | MK756570 | \\\ |
| 478 | *Pinanga adangensis* | XTBG028 | MK753927 | MK705038 | MK692321 | MK756986 | \\\ |
| 479 | *Pinanga adangensis* | XTBG034 | MK753928 | MK705039 | MK692322 | MK756987 | \\\ |
| 480 | *Pinanga baviensis* | SCBG142 | MK753935 | MK705049 | MK692318 | MK756975 | \\\ |
| 481 | *Pinanga baviensis* | XTBG024 | MK753936 | MK705050 | MK692319 | MK756976 | MK683342 |
| 482 | *Pinanga baviensis* | XTBG036 | MK753937 | MK705051 | MK692320 | MK756977 | MK683343 |
| 483 | *Pinanga coronata* | SCBG1000 | MK753931 | MK705042 | MK692325 | MK756980 | \\\ |
| 484 | *Pinanga coronata* | SCBG135 | MK753929 | MK705040 | MK692323 | MK756978 | \\\ |
| 485 | *Pinanga coronata* | SCBG969 | MK753930 | MK705041 | MK692324 | MK756979 | \\\ |
| 486 | *Pinanga coronata* | XTBG021 | MK753944 | MK705046 | MK692329 | \\\ | \\\ |
| 487 | *Pinanga coronata* | XTBG032 | MK753945 | MK705047 | MK692330 | \\\ | \\\ |
| 488 | *Pinanga coronata* | XTBG037 | MK753933 | MK705044 | MK692327 | MK756981 | \\\ |
| 489 | *Pinanga coronata* | XTBG194 | MK753932 | MK705043 | MK692326 | MK756982 | MK683346 |
| 490 | *Pinanga coronata* | XTBG212 | MK753934 | MK705045 | MK692328 | MK756983 | MK683347 |
| 491 | *Pinanga scortechinii* | SCBG143 | MK753946 | MK705048 | MK692331 | \\\ | \\\ |
| 492 | *Pinanga sylvestris* | XTBG026 | MK753949 | MK705036 | MK692332 | MK756984 | MK683344 |
| 493 | *Pinanga sylvestris* | XTBG035 | MK753950 | MK705037 | MK692333 | MK756985 | MK683345 |
| 494 | *Plectocomia elongata* | XTBG074 | MK754007 | MK705244 | MK692421 | MK757064 | MK683410 |
| 495 | *Plectocomia elongata* | XTBG077 | MK754008 | MK705245 | MK692422 | MK757065 | MK683411 |
| 496 | *Plectocomia pierreana* | XTBG075 | MK753996 | MK705246 | MK692423 | MK757059 | MK683412 |
| 497 | *Pritchardia pacifica* | XMBG008 | MK753873 | MK704623 | MK692005 | MK756512 | \\\ |
| 498 | *Pritchardia pacifica* | XMBG082 | MK753874 | MK704624 | MK692004 | MK756513 | \\\ |
| 499 | *Pritchardia pacifica* | XTBG015 | MK753609 | MK704625 | \\\ | MK756515 | \\\ |
| 500 | *Pritchardia schattaueri* | XMBG424 | MK753875 | MK704620 | \\\ | MK756514 | \\\ |
| 501 | *Pritchardia thurstonii* | XTBG123 | MK753610 | MK704626 | \\\ | MK756516 | \\\ |
| 502 | *Ptychosperma caryotoides* | SCBG043 | MK753657 | MK704976 | MK692188 | MK756843 | MK683257 |
| 503 | *Ptychosperma cuneatum* | XMBG134 | MK753659 | MK704978 | MK692190 | MK756834 | MK683258 |
| 504 | *Ptychosperma elegans* | SCBG144 | MK753674 | MK704992 | MK692205 | MK756835 | \\\ |
| 505 | *Ptychosperma elegans* | XMBG112 | MK753675 | MK704993 | MK692206 | MK756836 | \\\ |
| 506 | *Ptychosperma lineare* | XTBG061 | MK753666 | MK704985 | MK692198 | MK756845 | MK683262 |
| 507 | *Ptychosperma macarthurii* | SCBG005 | MK753660 | MK704979 | MK692191 | MK756846 | MK683273 |
| 508 | *Ptychosperma macarthurii* | SCBG889 | MK753661 | MK704980 | MK692192 | MK756837 | MK683271 |
| 509 | *Ptychosperma microcarpum* | XTBG039 | MK753662 | MK704981 | MK692193 | MK756838 | MK683263 |
| 510 | *Ptychosperma propinquum* | XMBG191 | MK753673 | MK704994 | MK692197 | MK756850 | MK683267 |
| 511 | *Ptychosperma pullenii* | SCBG003 | MK753663 | MK704982 | MK692194 | MK756872 | MK683259 |
| 512 | *Ptychosperma salomonense* | SCBG044 | MK753669 | MK704988 | MK692201 | MK756847 | MK683261 |
| 513 | *Ptychosperma salomonense* | SCBG884 | MK753671 | MK704990 | MK692203 | MK756839 | MK683264 |
| 514 | *Ptychosperma salomonense* | XTBG050 | MK753670 | MK704989 | MK692202 | MK756848 | MK683265 |
| 515 | *Ptychosperma salomonense* | XTBG051 | MK753672 | MK704991 | MK692204 | MK756840 | MK683266 |
| 516 | *Ptychosperma sanderianum* | XTBG128 | MK753667 | MK704986 | MK692199 | MK756841 | MK683268 |
| 517 | *Ptychosperma sp.* | SCBG004 | MK753668 | MK704987 | MK692200 | MK756842 | MK683260 |
| 518 | *Ptychosperma sp.* | SCBG006 | MK753664 | MK704983 | MK692195 | MK756849 | MK683269 |
| 519 | *Ptychosperma sp.* | XTBG057 | MK753658 | MK704977 | MK692189 | MK756844 | MK683272 |
| 520 | *Ptychosperma waitianum* | XTBG048 | MK753665 | MK704984 | MK692196 | MK756873 | MK683270 |
| 521 | *Raphia taedigera* | XTBG157 | MK754046 | MK705186 | \\\ | MK757004 | \\\ |
| 522 | *Raphia vinifera* | SCBG949 | MK754042 | MK705187 | MK692424 | MK757005 | \\\ |
| 523 | *Raphia vinifera* | XMBG031 | MK754045 | MK705190 | MK692427 | MK757006 | \\\ |
| 524 | *Raphia vinifera* | XMBG102 | MK754043 | MK705188 | MK692425 | MK757007 | \\\ |
| 525 | *Raphia vinifera* | XTBG067 | MK754044 | MK705189 | MK692426 | MK757008 | \\\ |
| 526 | *Ravenea dransfieldii* | SCBG166 | MK753480 | MK705016 | MK692121 | MK756742 | MK683096 |
| 527 | *Ravenea hildebrandtii* | SCBG072 | MK753481 | MK705017 | MK692124 | MK756743 | \\\ |
| 528 | *Ravenea rivularis* | SCBG029 | MK753482 | MK704837 | MK692122 | MK756744 | MK683097 |
| 529 | *Ravenea rivularis* | SCBG991 | MK753483 | MK704838 | MK692123 | MK756745 | \\\ |
| 530 | *Ravenea xerophila* | SCBG171 | MK753484 | MK704839 | MK692125 | MK756739 | MK683098 |
| 531 | *Ravenea xerophila* | XMBG215 | MK753485 | MK704840 | MK692126 | MK756740 | MK683099 |
| 532 | *Ravenea xerophila* | XMBG249 | MK753486 | MK704841 | MK692127 | MK756741 | MK683100 |
| 533 | *Reinhardtia gracilis* | SCBG060 | MK753851 | MK704749 | MK692131 | MK756966 | \\\ |
| 534 | *Reinhardtia gracilis* | XMBG414 | MK753852 | MK704750 | MK692132 | MK756967 | \\\ |
| 535 | *Reinhardtia paiewonskiana* | XMBG043 | MK753770 | MK704752 | MK692134 | MK756965 | \\\ |
| 536 | *Reinhardtia simplex* | SCBG179 | MK753853 | MK704751 | MK692133 | MK756968 | MK683338 |
| 537 | *Rhapidophyllum hystrix* | XTBG163 | MK753549 | MK704606 | MK691949 | MK756346 | MK683143 |
| 538 | *Rhapis cochinchinensis* | XTBG126 | MK753564 | MK704584 | MK692137 | MK756519 | \\\ |
| 539 | *Rhapis excelsa* | SCBG118 | MK753577 | MK704593 | MK692150 | MK756526 | \\\ |
| 540 | *Rhapis excelsa* | SCBG119 | MK753580 | MK704596 | MK692153 | MK756527 | \\\ |
| 541 | *Rhapis excelsa* | SCBG120 | MK753578 | MK704594 | MK692151 | MK756528 | \\\ |
| 542 | *Rhapis excelsa* | SCBG150 | MK753579 | MK704595 | MK692152 | MK756535 | \\\ |
| 543 | *Rhapis excelsa* | XMBG025 | MK753581 | MK704597 | MK692154 | MK756529 | \\\ |
| 544 | *Rhapis excelsa* | XTBG005 | MK753582 | MK704598 | MK692155 | MK756530 | \\\ |
| 545 | *Rhapis excelsa* | XTBG006 | MK753583 | MK704599 | MK692156 | MK756531 | \\\ |
| 546 | *Rhapis gracilis* | SCBG596 | MK753570 | MK704607 | MK692143 | MK756520 | \\\ |
| 547 | *Rhapis humilis* | SCBG157 | MK753573 | MK704590 | MK692146 | MK756532 | \\\ |
| 548 | *Rhapis humilis* | SCBG158 | MK753565 | MK704585 | MK692138 | MK756521 | MK683165 |
| 549 | *Rhapis humilis* | SCBG164 | MK753574 | MK704591 | MK692147 | MK756533 | MK683168 |
| 550 | *Rhapis humilis* | SCBG165 | MK753566 | MK704586 | MK692139 | MK756522 | MK683166 |
| 551 | *Rhapis humilis* | SCBG895 | MK753575 | MK704592 | MK692148 | MK756534 | MK683167 |
| 552 | *Rhapis multifida* | SCBG982 | MK753568 | MK704588 | MK692141 | MK756523 | \\\ |
| 553 | *Rhapis multifida* | XMBG409 | MK753569 | MK704589 | MK692142 | MK756524 | \\\ |
| 554 | *Rhapis sp.* | SCBG117 | MK753571 | MK704608 | MK692144 | MK756536 | \\\ |
| 555 | *Rhapis sp.* | SCBG964 | MK753572 | MK704609 | MK692145 | MK756537 | \\\ |
| 556 | *Rhapis sp.* | XTBG124 | MK753567 | MK704587 | MK692140 | MK756525 | \\\ |
| 557 | *Rhapis subtilis* | SCBG152 | MK753576 | MK704610 | MK692149 | MK756538 | \\\ |
| 558 | *Rhopaloblaste augusta* | XTBG088 | MK753394 | MK704967 | MK692298 | MK756950 | MK683325 |
| 559 | *Rhopalostylis sapida* | XMBG245 | MK753990 | MK764383 | MK692243 | MK756954 | MK683301 |
| 560 | *Roscheria melanochaetes* | SCBG996 | MK753896 | MK705029 | MK692256 | MK756989 | \\\ |
| 561 | *Roystonea borinquena* | XMBG192 | MK753865 | MK704870 | \\\ | MK756788 | \\\ |
| 562 | *Roystonea oleracea* | SCBG024 | MK753866 | MK704871 | \\\ | MK756789 | \\\ |
| 563 | *Roystonea oleracea* | XTBG171 | MK753867 | MK704872 | \\\ | MK756790 | \\\ |
| 564 | *Roystonea regia* | SCBG136 | MK753868 | MK704873 | \\\ | MK756791 | \\\ |
| 565 | *Roystonea sp.* | SCBG017 | MK753869 | MK704874 | \\\ | MK756792 | \\\ |
| 566 | *Sabal bermudana* | SCBG026 | MK753611 | MK704724 | MK692039 | MK756572 | MK683052 |
| 567 | *Sabal causiarum* | SCBG025 | MK753612 | MK704725 | MK692040 | MK756573 | MK683056 |
| 568 | *Sabal causiarum* | SCBG027 | MK753613 | MK704726 | MK692041 | MK756574 | MK683053 |
| 569 | *Sabal domingensis* | XTBG168 | MK753614 | MK704727 | MK692042 | MK756576 | \\\ |
| 570 | *Sabal etonia* | SCBG091 | MK753615 | MK704728 | MK692043 | MK756577 | MK683057 |
| 571 | *Sabal etonia* | XMBG407 | MK753616 | MK704729 | MK692044 | MK756578 | \\\ |
| 572 | *Sabal mauritiiformis* | SCBG030 | MK753628 | MK704743 | MK692056 | MK756591 | \\\ |
| 573 | *Sabal mauritiiformis* | XMBG063 | MK753630 | MK704745 | MK692058 | MK756592 | \\\ |
| 574 | *Sabal mauritiiformis* | XTBG111 | MK753629 | MK704744 | MK692057 | MK756593 | \\\ |
| 575 | *Sabal mexicana* | SCBG053 | MK753632 | MK704742 | MK692060 | MK756579 | \\\ |
| 576 | *Sabal mexicana* | SCBG012 | MK753631 | MK704741 | MK692059 | MK756575 | \\\ |
| 577 | *Sabal minor* | SCBG052 | MK753617 | MK704730 | MK692045 | MK756580 | \\\ |
| 578 | *Sabal minor* | SCBG096 | MK753618 | MK704731 | MK692046 | MK756581 | \\\ |
| 579 | *Sabal minor* | SCBG915 | MK753619 | MK704732 | MK692047 | MK756582 | \\\ |
| 580 | *Sabal minor* | XTBG107 | MK753620 | MK704733 | MK692048 | MK756583 | \\\ |
| 581 | *Sabal palmetto* | SCBG035 | MK753621 | MK704734 | MK692049 | MK756584 | MK683054 |
| 582 | *Sabal palmetto* | SCBG076 | MK753622 | MK704735 | MK692050 | MK756585 | \\\ |
| 583 | *Sabal palmetto* | SCBG092 | MK753623 | MK704736 | MK692051 | MK756586 | MK683058 |
| 584 | *Sabal rosei* | SCBG137 | MK753624 | MK704737 | MK692052 | MK756587 | MK683055 |
| 585 | *Sabal sp.* | SCBG067 | MK753625 | MK704738 | MK692053 | MK756588 | \\\ |
| 586 | *Sabal sp.* | XMBG072 | MK753626 | MK704739 | MK692054 | MK756589 | \\\ |
| 587 | *Sabal sp.* | XMBG425 | MK753627 | MK704740 | MK692055 | MK756590 | \\\ |
| 588 | *Salacca clemensiana* | XTBG150 | MK754047 | MK705236 | MK692428 | MK757051 | MK683413 |
| 589 | *Salacca clemensiana* | XTBG153 | MK754048 | MK705237 | MK692429 | MK757052 | MK683414 |
| 590 | *Salacca glabrescens* | XTBG154 | MK754051 | MK705240 | MK692432 | MK757061 | MK683416 |
| 591 | *Salacca glabrescens* | XTBG229 | MK754052 | MK705241 | MK692433 | MK757062 | MK683418 |
| 592 | *Salacca secunda* | XTBG040 | MK754037 | MK705243 | MK692435 | MK757060 | MK683419 |
| 593 | *Salacca wallichiana* | XTBG148 | MK754053 | MK705242 | MK692434 | MK757063 | MK683417 |
| 594 | *Salacca zalacca* | XMBG436 | MK754049 | MK705238 | MK692430 | MK757057 | \\\ |
| 595 | *Salacca zalacca* | XTBG065 | MK754050 | MK705239 | MK692431 | MK757058 | MK683415 |
| 596 | *Saribus rotundifolius* | SCBG009 | MK753454 | MK704642 | MK692007 | MK756459 | \\\ |
| 597 | *Saribus rotundifolius* | SCBG952 | MK753455 | MK704643 | MK692008 | MK756460 | \\\ |
| 598 | *Saribus rotundifolius* | SCBG972 | MK753458 | MK704646 | MK692011 | MK756462 | \\\ |
| 599 | *Saribus rotundifolius* | XMBG130 | MK753456 | MK704644 | MK692009 | MK756461 | \\\ |
| 600 | *Saribus rotundifolius* | XTBG162 | MK753457 | MK704645 | MK692010 | MK756463 | MK683152 |
| 601 | *Satakentia liukiuensis* | XTBG236 | MK753706 | MK704999 | MK692251 | MK756876 | MK683320 |
| 602 | *Schippia concolor* | SCBG199 | MK754056 | MK704721 | MK692036 | MK756635 | \\\ |
| 603 | *Schippia concolor* | SCBG945 | MK754057 | MK704722 | MK692037 | MK756636 | MK683088 |
| 604 | *Serenoa repens* | SCBG198 | MK753537 | MK704556 | MK691913 | MK756336 | MK683142 |
| 605 | *Serenoa repens* | XMBG095 | MK753538 | MK704557 | MK691914 | MK756338 | \\\ |
| 606 | *Serenoa repens* | XMBG182 | MK753539 | MK704558 | MK691915 | MK756337 | \\\ |
| 607 | *Serenoa repens* | XMBG235 | MK753540 | MK704559 | MK691916 | MK756754 | \\\ |
| 608 | *Syagrus amara* | SCBG031 | MK753800 | MK704788 | MK691869 | MK756359 | \\\ |
| 609 | *Syagrus amara* | SCBG033 | MK753801 | MK704789 | MK691870 | MK756360 | \\\ |
| 610 | *Syagrus amara* | SCBG082 | MK753802 | MK704790 | MK691871 | MK756361 | MK683104 |
| 611 | *Syagrus coronata* | SCBG022 | MK753818 | MK704806 | MK691881 | MK756366 | \\\ |
| 612 | *Syagrus coronata* | SCBG023 | MK753819 | MK704807 | MK691882 | MK756367 | MK683120 |
| 613 | *Syagrus coronata* | SCBG102 | MK753822 | MK704810 | MK691885 | MK756368 | \\\ |
| 614 | *Syagrus coronata* | XMBG041 | MK753817 | MK704805 | MK691880 | MK756355 | \\\ |
| 615 | *Syagrus coronata* | XTBG127 | MK753820 | MK704808 | MK691883 | MK756369 | \\\ |
| 616 | *Syagrus coronata* | XTBG129 | MK753821 | MK704809 | MK691884 | MK756356 | MK683107 |
| 617 | *Syagrus macrocarpa* | SCBG077 | MK753803 | MK704791 | MK691872 | MK756362 | \\\ |
| 618 | *Syagrus macrocarpa* | XMBG088 | MK753804 | MK704792 | MK691873 | MK756363 | \\\ |
| 619 | *Syagrus macrocarpa* | XTBG177 | MK753805 | MK704793 | MK691874 | MK756364 | MK683105 |
| 620 | *Syagrus picrophylla* | XMBG103 | MK753807 | MK704795 | MK691861 | MK756407 | \\\ |
| 621 | *Syagrus picrophylla* | XMBG104 | MK753808 | MK704796 | MK691862 | MK756403 | \\\ |
| 622 | *Syagrus picrophylla* | XMBG105 | MK753809 | MK704797 | MK691863 | MK756404 | \\\ |
| 623 | *Syagrus picrophylla* | XMBG106 | MK753810 | MK704798 | MK691864 | MK756405 | \\\ |
| 624 | *Syagrus picrophylla* | XMBG413 | MK753811 | MK704799 | MK691865 | MK756406 | MK683106 |
| 625 | *Syagrus romanzoffiana* | SCBG039 | MK753806 | MK704794 | MK691875 | MK756357 | \\\ |
| 626 | *Syagrus sancona* | SCBG032 | MK753815 | MK704803 | MK691876 | MK756414 | MK683110 |
| 627 | *Syagrus sancona* | XMBG113 | MK753816 | MK704804 | MK691877 | MK756491 | MK683109 |
| 628 | *Syagrus schizophylla* | XMBG108 | MK753812 | MK704800 | MK691866 | MK756400 | \\\ |
| 629 | *Syagrus schizophylla* | XMBG233 | MK753813 | MK704801 | MK691867 | MK756401 | MK683111 |
| 630 | *Syagrus schizophylla* | XMBG417 | MK753814 | MK704802 | MK691868 | MK756402 | MK683121 |
| 631 | *Syagrus weddelliana* | SCBG965 | MK753823 | MK704811 | MK691887 | MK756693 | \\\ |
| 632 | *Syagrus weddelliana* | XMBG055 | MK753825 | MK704823 | MK691889 | MK756370 | \\\ |
| 633 | *Syagrus weddelliana* | XMBG306 | MK753824 | MK704812 | MK691888 | MK756365 | MK683108 |
| 634 | *Thrinax excelsa* | SCBG063 | MK753602 | MK704701 | MK692076 | MK756617 | MK683085 |
| 635 | *Thrinax excelsa* | XMBG056 | MK753603 | MK704702 | MK692077 | MK756618 | MK683087 |
| 636 | *Thrinax parviflora* | SCBG036 | MK753604 | MK704703 | MK692078 | MK756619 | MK683086 |
| 637 | *Thrinax radiata* | XMBG242 | MK753605 | MK704704 | MK692079 | MK756620 | \\\ |
| 638 | *Thrinax sp.* | XMBG244 | MK753606 | MK704705 | MK692080 | MK756621 | \\\ |
| 639 | *Trachycarpus fortunei* | XMBG057 | MK753550 | MK704601 | MK691951 | MK756340 | \\\ |
| 640 | *Trachycarpus fortunei* | XMBG058 | MK753551 | MK704602 | MK691952 | MK756341 | \\\ |
| 641 | *Trachycarpus fortunei* | XMBG107 | MK753552 | MK704603 | MK691953 | MK756342 | \\\ |
| 642 | *Trachycarpus fortunei* | XTBG169 | MK753553 | MK704604 | MK691954 | MK756343 | MK683145 |
| 643 | *Trachycarpus martianus* | SCBG947 | MK753555 | MK704605 | MK691957 | MK756413 | \\\ |
| 644 | *Trachycarpus martianus* | XMBG110 | MK753556 | MK704618 | MK691958 | MK756345 | MK683150 |
| 645 | *Trachycarpus martianus* | XTBG166 | MK753563 | MK704617 | MK691959 | \\\ | \\\ |
| 646 | *Trachycarpus nanus* | XTBG167 | MK753554 | MK704616 | MK691956 | MK756475 | MK683146 |
| 647 | *Trithrinax brasiliensis var. acanthocoma* | SCBG114 | MK753876 | MK704715 | MK692071 | MK756631 | \\\ |
| 648 | *Trithrinax brasiliensis var. acanthocoma* | SCBG116 | MK753877 | MK704716 | MK692072 | MK756632 | \\\ |
| 649 | *Trithrinax brasiliensis var. acanthocoma* | SCBG944 | MK753878 | MK704717 | MK692073 | MK756633 | \\\ |
| 650 | *Trithrinax brasiliensis var. acanthocoma* | XMBG234 | MK753607 | MK704720 | MK692074 | MK756630 | \\\ |
| 651 | *Trithrinax brasiliensis* | XMBG415 | MK753880 | MK704719 | MK692075 | MK756628 | \\\ |
| 652 | *Trithrinax campestris* | SCBG080 | MK753879 | MK704718 | MK692070 | MK756629 | \\\ |
| 653 | *Veitchia arecina* | XTBG042 | MK753646 | MK705069 | MK692177 | MK756917 | MK683247 |
| 654 | *Veitchia arecina* | XTBG085 | MK753647 | MK705070 | MK692178 | MK756918 | MK683248 |
| 655 | *Veitchia arecina* | XTBG196 | MK753648 | MK705071 | MK692179 | MK756919 | MK683254 |
| 656 | *Veitchia filifera* | XTBG041 | MK753650 | MK705064 | MK692181 | MK756916 | MK683252 |
| 657 | *Veitchia joannis* | SCBG088 | MK753651 | MK705065 | MK692182 | MK756810 | MK683251 |
| 658 | *Veitchia sp.* | SCBG162 | MK753652 | MK705066 | MK692183 | MK756913 | MK683253 |
| 659 | *Veitchia spiralis* | SCBG087 | MK753653 | MK705067 | MK692184 | MK756914 | MK683249 |
| 660 | *Veitchia vitiensis* | XMBG253 | MK753649 | MK705072 | MK692180 | MK756920 | MK683246 |
| 661 | *Veitchia winin* | XMBG180 | MK753654 | MK705073 | MK692185 | MK756921 | MK683250 |
| 662 | *Verschaffeltia splendida* | XTBG102 | MK753395 | MK705030 | MK692375 | MK757068 | MK683326 |
| 663 | *Washingtonia filifera* | SCBG881 | MK753543 | MK704568 | MK691999 | MK756565 | MK683186 |
| 664 | *Washingtonia filifera* | SCBG893 | MK753544 | MK704569 | MK692000 | MK756566 | \\\ |
| 665 | *Washingtonia filifera* | XMBG001 | MK753545 | MK704570 | MK692001 | MK756567 | MK683187 |
| 666 | *Wodyetia bifurcata* | SCBG034 | MK753676 | MK704945 | MK692218 | MK756864 | MK683286 |
| 667 | *Wodyetia bifurcata* | SCBG075 | MK753678 | MK704947 | MK692220 | MK756865 | MK683287 |
| 668 | *Wodyetia bifurcata* | SCBG154 | MK753677 | MK704946 | MK692219 | MK756866 | MK683288 |
| 669 | *Zombia antillarum* | XTBG216 | MK753599 | MK704700 | MK692032 | MK756616 | \\\ |
