# Supplementary material for: The utility of DNA barcodes to confirm the identification of palm collections in botanical gardens
Source: PLoS One. 2020 Jul 31;15(7):e0235569. doi: 10.1371/journal.pone.0235569 (PMC7394517; doi:10.1371/journal.pone.0235569)
Supplement: S2 Table — (DOCX) [file pone.0235569.s002.docx]

**Table S2.** The species resolution of the eight large genera studied (≥ 5 species per genus)

Persentage (%)

| **Genus** | **No. of Species** | **R** | **M** | **I** | **I2** | **T** | **MR** | **I2R** | **RT** | **I2M** | **MT** | **I2T** | **I2MR** | **I2MT** | **MRT** | **I2RT** | **I2MRT** |
| --- | --- | --- | --- | --- | --- | --- | --- | --- | --- | --- | --- | --- | --- | --- | --- | --- | --- |
| *Arenga* | 6 | 0.0 | 0.0 | 60.0 | 50.0 | 16.7 | 16.7 | 50.0 | 16.7 | 50.0 | 33.3 | 33.3 | 66.7 | 33.3 | 33.3 | 33.3 | 33.3 |
| *Calamus* | 11 | 36.4 | 54.5 | 28.6 | 72.7 | 72.7 | 72.7 | 81.8 | 90.9 | 81.8 | 81.8 | 90.9 | 90.9 | 90.9 | 90.9 | 100.0 | 100.0 |
| *Chamaedorea* | 7 | 28.6 | 85.7 | 100.0 | 100.0 | 42.9 | 85.7 | 100.0 | 57.1 | 100.0 | 85.7 | 100.0 | 100.0 | 100.0 | 85.7 | 100.0 | 100.0 |
| *Dypsis* | 10 | 20.0 | 40.0 | 80.0 | 80.0 | 40.0 | 70.0 | 80.0 | 40.0 | 90.0 | 40.0 | 60.0 | 90.0 | 60.0 | 50.0 | 60.0 | 60.0 |
| *Livistona* | 5 | 0.0 | 20.0 | 0.0 | 80.0 | 20.0 | 20.0 | 100.0 | 20.0 | 100.0 | 20.0 | 80.0 | 100.0 | 80.0 | 20.0 | 80.0 | 80.0 |
| *Phoenix* | 9 | 0.0 | 33.3 | 50.0 | 44.4 | 22.2 | 33.3 | 44.4 | 22.2 | 66.7 | 33.3 | 44.4 | 66.7 | 66.7 | 33.3 | 44.4 | 66.7 |
| *Sabal* | 6 | 0.0 | 0.0 | 0.0 | 33.3 | 16.7 | 0.0 | 33.3 | 16.7 | 33.3 | 16.7 | 33.3 | 33.3 | 33.3 | 16.7 | 33.3 | 33.3 |
| *Syagrus* | 7 | 0.0 | 0.0 | 66.7 | 100.0 | 14.3 | 0.0 | 100.0 | 14.3 | 100.0 | 14.3 | 85.7 | 100.0 | 85.7 | 14.3 | 85.7 | 85.7 |

*Note: “**No. of Species”** is number of species with multiple individuals. *rbcL* (R); *matK* (M); ITS (I); ITS2 (I2); *trnH-psbA* (T).
