# Supplementary material for: The utility of DNA barcodes to confirm the identification of palm collections in botanical gardens
Source: PLoS One. 2020 Jul 31;15(7):e0235569. doi: 10.1371/journal.pone.0235569 (PMC7394517; doi:10.1371/journal.pone.0235569)
Supplement: S3 Table — (DOCX) [file pone.0235569.s003.docx]

|  | **Table S3.** Misidentified samples in the botanical garden | | |
| --- | --- | --- | --- |
| **No.** | **Names identified in the botanical garden** | **Names confirmed** | **Voucher specimen** |
| 1 | *Ptychosperma salomonense* | *Ptychosperma elegans* | PSC144 |
| 2 | *Rhapis excelsa* | *Rhapis humilis* | PSC895 |
| 3 | *Aiphanes hirsuta* | *Aiphanes eggersii* | PXM175 |
| 4 | *Phoenix sylvestris* | *Phoenix andamanensis* | XTBG116 |
| 5 | *Ptychosperma sanderiana* | *Ptychosperma sp.* | PSC004 |
| 6 | *Ptychosperma hospitum* | *Ptychosperma macarthurii* | PSC005 |
| 7 | *Ptychosperma macarthurii* | *Ptychosperma sp.* | PSC006 |
| 8 | *Bactris mexicana* | *Bactris major* | PSC013 |
| 9 | *Bactris militaris* | *Bactris gasipaes* | PSC018 |
| 10 | *Sabal maritima* | *Sabal causiarum* | PSC027 |
| 11 | *Sabal causiarum* | *Sabal palmetto* | PSC035 |
| 12 | *Ptychosperma schefferi* | *Ptychosperma salomonense* | PSC044 |
| 13 | *Phoenix dactylifera* | *Phoenix atlantica* | PSC054 |
| 14 | *Sabal mauritiiformis* | *Sabal sp.* | PSC067 |
| 15 | *Astrocaryum mexicanum* | *Astrocaryum murumuru* | PSC073 |
| 16 | *Syagrus smithii* | *Syagrus macrocarpa* | PSC077 |
| 17 | *Attalea phalerata* | *Attalea oleifera* | PSC084 |
| 18 | *Sabal bermudana* | *Sabal etonia* | PSC091 |
| 19 | *Dypsis nodifera* | *Dypsis cabadae* | PSC101 |
| 20 | *Guihaia argyrata* | *Guihaia grossifibrosa* | PSC105 |
| 21 | *Calamus rhabdocladus* | *Calamus sp.* | PSC107 |
| 22 | *Dypsis baronii* | *Dypsis carlsmithii* | PSC139 |
| 23 | *Rhapis filiformis* | *Rhapis humilis* | PSC157 |
| 24 | *Veitchia arecina* | *Veitchia sp.* | PSC162 |
| 25 | *Rhapis excelsa* | *Rhapis humilis* | PSC164 |
| 26 | *Borassus flabellifer* | *Borassus madagascariensis* | PSC173 |
| 27 | *Reinhardtia gracilis* | *Reinhardtia simplex* | PSC179 |
| 28 | *Borassus flabellifer* | *Borassus aethiopum* | PSC849 |
| 29 | *Lanonia hainamensis* | *Lanonia centralis* | PSC877 |
| 30 | *Washingtonia robusta* | *Washingtonia filifera* | PSC881 |
| 31 | *Livistona speciosa* | *Livistona jenkinsiana* | PSC882 |
| 32 | *Attalea butyracea* | *Attalea amygdalina* | PSC899 |
| 33 | *Attalea cohune* | *Attalea amygdalina* | PSC948 |
| 34 | *Saribus merrillii* | *Saribus rotundifolius* | PSC952 |
| 35 | *Arenga hookeriana* | *Arenga caudata* | PSC961 |
| 36 | *Livistona drudei* | *Livistona decora* | PSC971 |
| 37 | *Rhapis humilis* | *Rhapis multifida* | PSC982 |
| 38 | *Chamaedorea seifrizii* | *Chamaedorea anemophila* | PSC986 |
| 39 | *Livistona saribus* | *Livistona speciosa* | PSC993 |
| 40 | *Livistona decora* | *Livistona drudei* | PSC994 |
| 41 | *Copernicia alba* | *Copernicia baileyana* | PXM027 |
| 42 | *Elaeis oleifera* | *Elaeis guineensis* | PXM037 |
| 43 | *Astrocaryum aculeatum* | *Astrocaryum alatum* | PXM040 |
| 44 | *Wallichia gracilis* | *Arenga disticha* | PXM054 |
| 45 | *Trachycarpus takil* | *Trachycarpus fortunei* | PXM057 |
| 46 | *Attalea rostrata* | *Attalea phalerata* | PXM076 |
| 47 | *Cryosophila warscewiczii* | *Cryosophila guagara* | PXM080 |
| 48 | *Caryota urens* | *Caryota obtusa* | PXM087 |
| 49 | *Aiphanes aculeata* | *Aiphanes sp.* | PXM101 |
| 50 | *Bactris gasipaes* | *Bactris setulosa* | PXM117 |
| 51 | *Ravenea glauca* | *Ravenea xerophila* | PXM215 |
| 52 | *Trithrinax brasiliensis* | *Trithrinax acanthocoma* | PXM234 |
| 53 | *Licuala paludosa* | *Licuala ramsayi* | PXM246 |
| 54 | *Veitchia arecina* | *Veitchia vitiensis* | PXM253 |
| 55 | *Lytocaryum insigne* | *Syagrus weddellianum* | PXM306 |
| 56 | *Brahea brandegeei* | *Brahea aculeata* | PXM411 |
| 57 | *Burretiokentia vieillardii* | *Burretiokentia hapala* | PXM438 |
| 58 | *Attalea rostrata* | *Attalea amygdalina* | XTBG003 |
| 59 | *Phoenix loureiroi* | *Phoenix roebelenii* | XTBG007 |
| 60 | *Phoenix loureiroi* | *Phoenix roebelenii* | XTBG008 |
| 61 | *Phoenix loureiroi* | *Phoenix pusilla* | XTBG010 |
| 62 | *Pinanga sylvestris* | *Pinanga baviensis* | XTBG024 |
| 63 | *Arenga australasica* | *Arenga tremula* | XTBG029 |
| 64 | *Pinanga coronata* | *Pinanga adangensis* | XTBG034 |
| 65 | *Pinanga baviensis* | *Pinanga sylvestris* | XTBG035 |
| 66 | *Pinanga gracilis* | *Pinanga baviensis* | XTBG036 |
| 67 | *Ptychosperma macarthurii* | *Ptychosperma salomonense* | XTBG051 |
| 68 | *Borassus aethiopum* | *Borassus madagascariensis* | XTBG058 |
| 69 | *Caryota cumingii* | *Caryota mitis* | XTBG066 |
| 70 | *Calamus nambariensis* | *Calamus palustris* | XTBG072 |
| 71 | *Daemonorops jenkinsiana* | *Calamus erectus* | XTBG076 |
| 72 | *Arenga obtusifolia* | *Arenga australasica* | XTBG083 |
| 73 | *Veitchia simulans* | *Veitchia arecina* | XTBG085 |
| 74 | *Dypsis fibrosa* | *Dypsis utilis* | XTBG094 |
| 75 | *Brassiophoenix schumannii* | *Brassiophoenix drymophloeoides* | XTBG118 |
| 76 | *Rhapis cochinchinensis* | *Rhapis sp.* | XTBG124 |
| 77 | *Syagrus oleracea* | *Syagrus coronata* | XTBG129 |
| 78 | *Calamus nambariensis* | *Calamus palustris* | XTBG137 |
| 79 | *Calamus caesius* | *Calamus exilis* | XTBG141 |
| 80 | *Salacca zalacca* | *Salacca clemensiana* | XTBG153 |
| 81 | *Salacca zalacca* | *Salacca glabrescens* | XTBG154 |
| 82 | *Calamus tetradactylus* | *Calamus floribundus* | XTBG155 |
| 83 | *Attalea speciosa* | *Attalea oleifera* | XTBG159 |
| 84 | *Copernicia baileyana* | *Copernicia hospita* | XTBG164 |
| 85 | *Arenga hookeriana* | *Arenga caudata* | XTBG176 |
| 86 | *Arenga hookeriana* | *Arenga caudata* | XTBG179 |
| 87 | *Calamus palustris* | *Calamus nambariensis* | XTBG182 |
| 88 | *Salacca zalacca* | *Salacca glabrescens* | XTBG229 |
| 89 | *Euterpe oleracea* | *Euterpe precatoria* | XTBG230 |
| 90 | *Ptychosperma propinquum* | *Ptychosperma pullenii* | PSC003 |
| 91 | *Gaussia maya* | *Cyphophoenix alba* | PSC011 |
| 92 | *Licuala* | *Lanonia centralis* | PSC020 |
| 93 | *Chamaedorea* | *Dypsis boiviniana* | PSC094 |
| 94 | *Ravenea robustior* | *Beccariophoenix fenestralis* | PSC098 |
| 95 | *Arenga tremula* | *Butia capitata* | PSC875 |
| 96 | *Licuala* | *Lanonia dasyantha* | PSC890 |
| 97 | *Livistona mariae* | *Saribus rotundifolius* | PSC972 |
| 98 | *Thrinax morrisii* | *Leucothrinax morrisii* | PXM047 |
| 99 | *Kentiopsis* | *Dypsis rivularis* | PXM092 |
| 100 | *Victyosperma album* | *Nannorrhops ritchieana* | PXM254 |
| 101 | *Licuala robinsoniana* | *Lanonia centralis* | XTBG025 |
| 102 | *Licuala mattanensis* | *Lanonia dasyantha* | XTBG031 |
| 103 | *Hydriastele* | *Balaka seemannii* | XTBG053 |
| 104 | *Arenga obtusifolia* | *Caryota monostachya* | XTBG064 |
| 105 | *Licuala mattanensis* | *Lanonia dasyantha* | XTBG105 |
| 106 | *Lanonia sp.* | *Licuala paludosa* | XTBG106 |
| 107 | *Hydriastele microcarpa* | *Calyptrocalyx elegans* | XTBG119 |
| 108 | *Cyphosperma* | *Nephrosperma van-houtteanum* | XTBG181 |
| 109 | *Hydriastele* | *Balaka seemannii* | XTBG191 |
| 110 | *Hydriastele* | *Balaka seemannii* | XTBG207 |
