# Supplementary figures and images for: The utility of DNA barcodes to confirm the identification of palm collections in botanical gardens
Source: PLoS One. 2020 Jul 31;15(7):e0235569. doi: 10.1371/journal.pone.0235569 (PMC7394517; doi:10.1371/journal.pone.0235569)

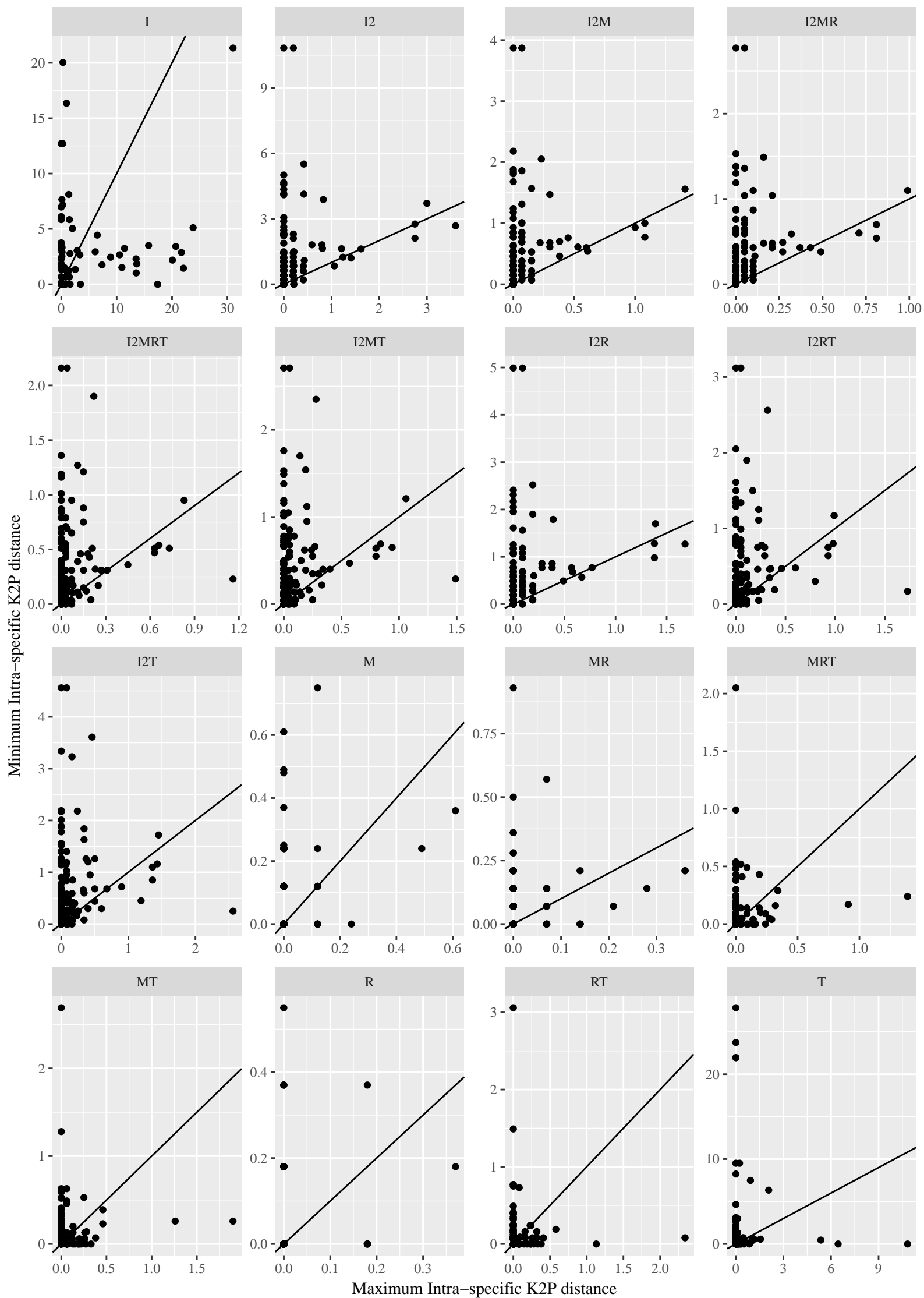

Supplement: S1 Fig — (PDF) [file pone.0235569.s004.pdf]

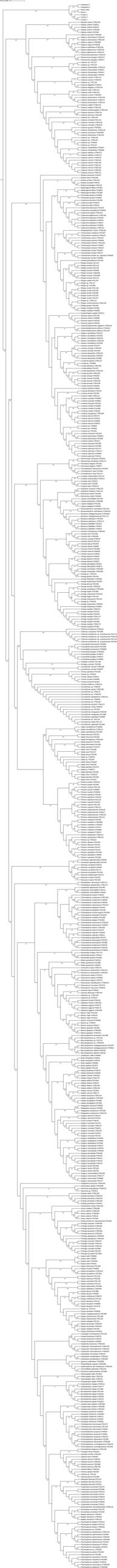

Supplement: S4 Fig — Bootstrap values are shown below the relevant branches. (PDF) [file pone.0235569.s007.pdf]
